# Repeated antibiotic exposure and risk of hospitalisation and death following COVID-19 infection (OpenSAFELY): a matched case–control study

**DOI:** 10.1016/j.eclinm.2023.102064

**Published:** 2023-07-05

**Authors:** Ya-Ting Yang, David Wong, Darren M. Ashcroft, Jon Massey, Brian MacKenna, Louis Fisher, Amir Mehrkar, Sebastian CJ. Bacon, the OpenSAFELY collaborative, Kieran Hand, Xiaomin Zhong, Ali Fahmi, Ben Goldacre, Tjeerd van Staa, Victoria Palin

**Affiliations:** a Centre for Health Informatics, School of Health Sciences, Faculty of Biology, Medicine, and Health, University of Manchester, Manchester, UK; b Leeds Institute of Health Sciences, School of Medicine, University of Leeds, Leeds, UK; c Centre for Pharmacoepidemiology and Drug Safety, School of Health Sciences, Faculty of Biology, Medicine and Health, University of Manchester, Manchester, UK; d NIHR Greater Manchester Patient Safety Translational Research Centre, School of Health Sciences, Faculty of Biology, Medicine and Health, University of Manchester, Manchester, UK; e Bennett Institute for Applied Data Science, Nuffield Department of Primary Care Health Sciences, University of Oxford, Oxford, UK; f NHS England, London, UK; g Division of Developmental Biology and Medicine, Maternal and Fetal Research Centre, University of Manchester, St Marys Hospital, Manchester, UK

**Keywords:** Antibiotics, Primary care, COVID-19, Severe outcome

## Abstract

**Background:**

Identifying potential risk factors related to severe COVID-19 outcomes is important. Repeated intermittent antibiotic use is known be associated with adverse outcomes. This study aims to examine whether prior frequent antibiotic exposure is associated with severe COVID-19 outcomes.

**Methods:**

With the approval of NHS England, we used the OpenSAFELY platform, which integrated primary and secondary care, COVID-19 test, and death registration data. This matched case–control study included 0.67 million patients (aged 18–110 years) from an eligible 2.47 million patients with incident COVID-19 by matching with replacement. Inclusion criteria included registration within one general practice for at least 3 years and infection with incident COVID-19. Cases were identified according to different severity of COVID-19 outcomes. Cases and eligible controls were 1:6 matched on age, sex, region of GP practice, and index year and month of COVID-19 infection. Five quintile groups, based on the number of previous 3-year antibiotic prescriptions, were created to indicate the frequency of prior antibiotic exposure. Conditional logistic regression used to compare the differences between case and control groups, adjusting for ethnicity, body mass index, comorbidities, vaccination history, deprivation, and care home status. Sensitivity analyses were done to explore potential confounding and the effects of missing data.

**Findings:**

Based on our inclusion criteria, between February 1, 2020 and December 31, 2021, 98,420 patients were admitted to hospitals and 22,660 died. 55 unique antibiotics were prescribed. A dose–response relationship between number of antibiotic prescriptions and risk of severe COVID-19 outcome was observed. Patients in the highest quintile with history of prior antibiotic exposure had 1.80 times greater odds of hospitalisation compared to patients without antibiotic exposure (adjusted odds ratio [OR] 1.80, 95% Confidence Interval [CI] 1.75–1.84). Similarly, the adjusted OR for hospitalised patients with death outcomes was 1.34 (95% CI 1.28–1.41). Larger number of prior antibiotic type was also associated with more severe COVID-19 related hospital admission. The adjusted OR of quintile 5 exposure (the most frequent) with more than 3 antibiotic types was around 2 times larger than quintile 1 (only 1 type; OR 1.80, 95% CI 1.75–1.84 vs. OR 1.03, 95% CI 1.01–1.05).

**Interpretation:**

Our observational study has provided evidence that antibiotic exposure frequency and diversity may be associated with COVID-19 severity, potentially suggesting adverse effects of repeated intermittent antibiotic use. Future work could work to elucidate causal links and potential mechanisms. Antibiotic stewardship should put more emphasis on long-term antibiotic exposure and its adverse outcome to increase the awareness of appropriate antibiotics use.

**Funding:**

Health Data Research UK and 10.13039/501100000272National Institute for Health Research.

**Research in context**

**Evidence before this study**

Accumulating research indicates that repeated intermittent antibiotic use is associated with adverse outcomes, yet few research examined its effect on COVID-19. Patients with long-term exposure to more antibiotics might be at higher risk of infection-related complications or autoimmune disease compared to those with less antibiotics. Possible explanations might include antibiotic resistance or antibiotic perturbation of gut microbiota, which has been reported in relation to immune and metabolism regulation. Current Research indicated that SARS-CoV-2 virus infected patients through both respiratory and gastrointestinal tracts and then possibly induced later serious immune response depends on individual conditions or characteristics. Some clinical data also found that the composition of gut microbiome was altered in COVID-19 patients, especially for those exposed to antibiotic treatment. It is, therefore, hypothesised that patients who use antibiotics frequently may be more susceptible to COVID-19 infection.

**Added value of this study**

In our case–control study, patients with more frequent antibiotic exposure in the past 3 years were at higher odds to experience severe COVID-19 outcome, including hospital admission and 30-day mortality. In addition, the diversity of antibiotic type use was associated with COVID-19 hospital admission. It seems advisable to discourage the regular practice of indiscriminately prescribing antibiotics, repeatedly and intermittently, given their uncertain benefit and likely risks.

**Implications of all the available evidence**

Our observational findings have provided evidence to suggest that antibiotic exposure frequency and diversity may be associated with COVID-19 severity. Although no causal links can be determined, these data suggest potential adverse effects of repeated intermittent antibiotic use. Future work could work to elucidate causal links and potential mechanisms. Antibiotic stewardship efforts should put more emphasis on long-term antibiotic exposure and its adverse outcome to increase the awareness of appropriate antibiotics use.

## Introduction

The pandemic caused by the SARS-CoV-2 virus overwhelmed the world since March 2020. To date, the World Health Organization reports that there have been 533 million confirmed cases and 6 million deaths globally.^1^ In the UK, 22 million cases led to 196,000 deaths.^2^ The Coronavirus disease 2019 (COVID-19) can have different outcomes depending on patient characteristics. For most healthy people, COVID-19-related symptoms are mild, but older age, non-white ethnicity, and presence of comorbidities are associated with developing more severe symptoms and increased risk of COVID-19 related death.^3^, ^4^, ^5^, ^6^ It is important to understand what factors are associated with more severe COVID-19, including prior medication use. Observational studies have found that long-term history of oral corticosteroid use was associated with increased risk of death, while non-steroidal anti-inflammatory drug use (NSAIDs) was not.^4^^,^^7^ Research evidence is essential for supporting clinical treatment decisions in pandemics.

Repeated intermittent antibiotic use is known be associated with adverse outcomes. A dose–response relationship has been reported in previous studies, where a higher frequency of prior antibiotic exposure was associated with increased risk of infection-related complications and autoimmune disease.^8^^,^^9^ One potential explanation may be that frequent antibiotic use increases the likelihood of patients becoming colonised and infected with antibiotic-resistant pathogens, leading to antibiotic treatment failure and increased susceptibility to adverse consequences of infection. Other studies also pointed out that antibiotic treatment might alter gut microbiota, which can impact metabolic and immune function.^10^ While in most situations, gut microbiota will recover to baseline within a few weeks or even months after stopping an antibiotic course, frequent antibiotic perturbation may affect the resilience of gut microbiomes.^11^^,^^12^

In the UK, 71.4% of all antibiotics are prescribed in primary care settings.^13^ However, most antibiotic research in the COVID-19 pandemic focused on bacterial co-infections for COVID-19 patients admitted to hospital. No studies have explored how the severity of COVID-19 disease is affected by prior antibiotic use. Therefore, this population-based case–control study aimed to determine the relationship between prior antibiotic use and clinical outcomes for COVID-19 patients.

## Methods

### Data sources

On behalf of NHS England, we used primary care records form OpenSAFELY-TPP, which comprises nearly 22 million patients' electronic health records (EHRs) covering 40% of the population in England. They contain information about demographics, diagnosis and medications. These EHRs were linked, at patient-level, to the following databases: (1) SARS-CoV-2 PCR testing results from the Second Generation Surveillance System (SGSS), (2) Hospital admission data from the NHS Digital Secondary Use Service (SUS): part of Hospital Episode Statistics (HES), providing information about admissions, diagnosis, and treatments for discharged patients, (3) COVID-19 inpatient death data from the COVID-19 Patient Notification System (CPNS), and (4) death registration data from the Office for National Statistics (ONS). Data linkage was provided via the OpenSAFELY integrated platform, which is governed by National Health Service (NHS) England.

### Ethics

This study was approved by the Health Research Authority and NHS Research Ethics Committee [REC reference 21/SC/0287] (Supplementary Protocol).

### Study design

This population-based matched case–control study was divided into two parts. Study 1 evaluated hospital admission among COVID-19 patients identified from the general population. To evaluate the severity among cases from study 1 with COVID-19 related hospitalisation, study 2 evaluated severe outcome by measuring death.

### Case-control study 1

**Fig. 1:**
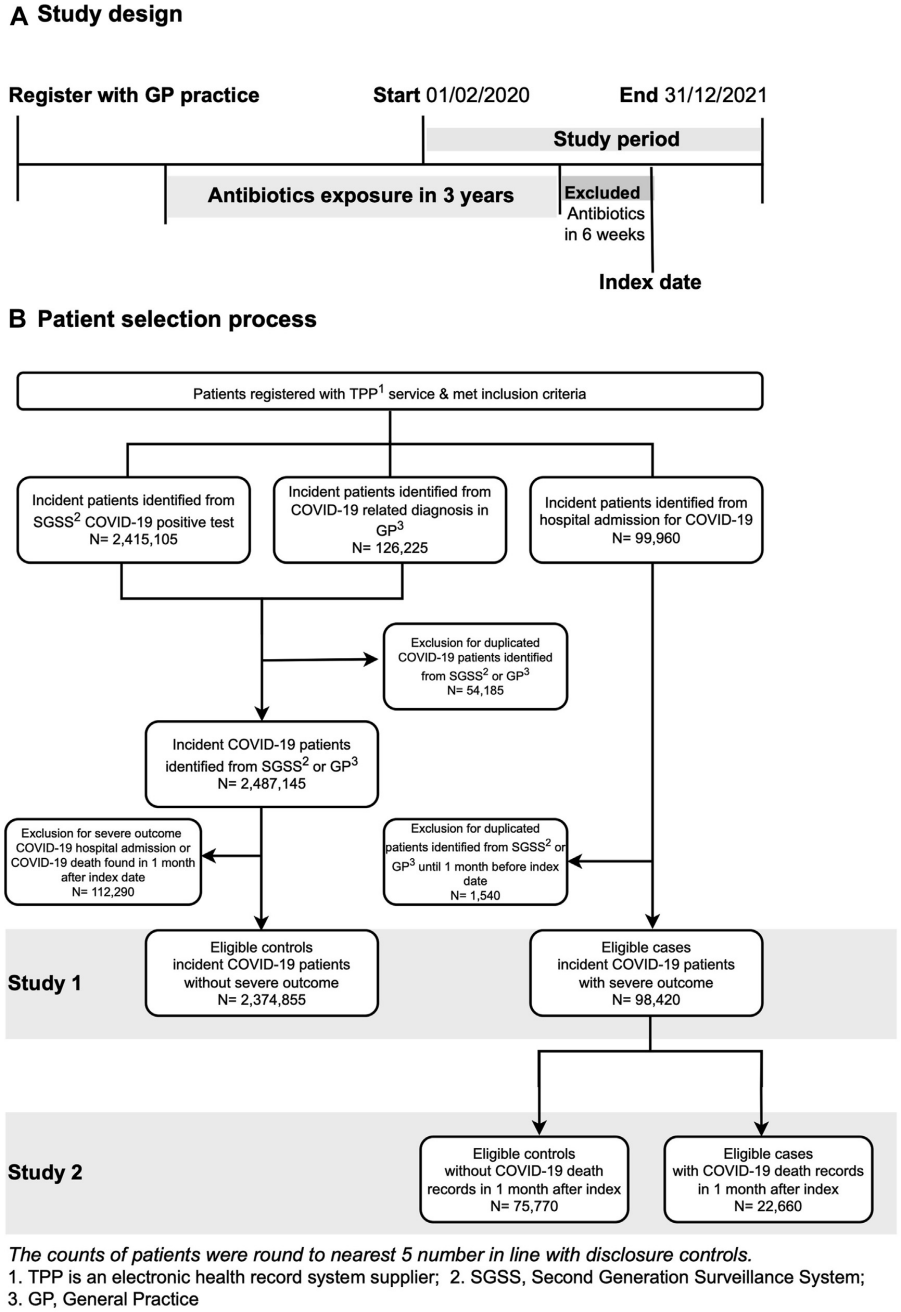
**Study design (A) andpatient selection (B).**

Eligible patients were selected from the beginning of the pandemic (01-02-2020), until the end of the year 2021 (31-12-2021) (Fig. 1A). Inclusion criteria consisted of: (1) complete age, sex, and region information; (2) aged 18–110 years; (3) registered with one practice for at least 3 years before the index date for a more complete prior history of antibiotics exposure; (4) incident COVID-19 infection identified from SGSS or coded in general practices (GP) in primary care and SUS in secondary care. Repeat COVID-19 records within 1 month were regarded as the same infection episode and the delineation of study design was in Supplementary Figure S1. For the small number of patients with more than one COVID-19 positive result (> one month apart), the first episode was used in this analysis and subsequent episodes excluded as the main focus was to investigate the effect of prior antibiotic exposure in patients with first exposure to COVID-19.

Cases were defined as patients admitted to hospital for COVID-19 with a primary diagnosis of International Classification of Diseases, 10th revision (ICD-10) codes U07.1 and U07.2. Controls were patients who had either COVID-19 positive test results or COVID-19 related diagnosis. The index date of cases was the incident date of COVID-19 hospital admission from SUS, while controls were identified from first COVID-19 record in GP records or confirmed positive test from SGSS. Prior COVID-19 history was searched from all databases and excluded for analysis. The flowchart of patient selection is illustrated in Fig. 1B. Since controls were limited to patients with minor COVID-19 clinical outcomes, patients with hospital admission or death records identified from SUS, CPNS or ONS within 1 month of index date were excluded.

### Case-control study 2

All patients hospitalised for COVID-19 in study 1 were selected for study 2 (Fig. 1B). Cases were patient with death certificate record (from ONS and CPNS) of COVID-19 related death within 1 month following admission. All other admitted patients were considered controls.

### Matching

For each study, cases were matched to up to 6 controls on age (within a maximum of 5 years), sex, region of general practices (NHS England regions divided by 44 Sustainability and Transformation Plan areas) to account for variation in infection prevalence, along with calendar year and month of the index date. The study inclusion period was divided into 3 waves according to index date, which were Wave 1 (early pandemic and first national lockdown, limited COVID-19 test availability for healthcare workers and severe symptoms patients): February–August 2020; Wave 2 (second national lockdown, COVID-19 availability for wider population): September 2020–April 2021; Wave 3 (end of national lockdown): May–December 2022.^14^^,^^15^ The distribution was shown in Table 1. To achieve comparable groups, matching with replacement was performed due to limited potential control pool for patients with COVID-19 (via R package MatchIT v4.2.0).^16^

**Table 1:** Characteristics of study cohorts before and after matching.

	Study 1: admitted to hospitals							
	Before matching				Matched			
	Case		Control		Case		Control	
	*n*^a^	%	*n*^a^	%	*n*^a^	%	*n*^a^	%
**Number of patients**	98,420	4.0	2,374,855	96.0	97,880	14.6	576,640	85.5
**Inclusion time**^b^								
Wave 1	20,295	20.6	65,680	2.8	19,880	20.3	111,605	19.4
Wave 2	50,375	51.2	781,415	32.9	50,295	51.4	299,675	52.0
Wave 3	27,750	28.2	1,527,760	64.3	27,705	28.3	165,360	28.7
**Sex**								
Male	54,600	55.5	1,098,125	46.2	54,270	55.4	318,360	55.2
Female	43,825	44.5	1,276,730	53.8	43,610	44.6	258,280	44.8
**Mean age (SD)**	65.7 (17.7)		43.3 (15.8)		65.6 (17.6)		65.2 (17.5)	
**Age group**								
18–29	2880	2.9	541,030	22.8	2880	2.9	17,270	3.0
30–39	6290	6.4	486,925	20.5	6285	6.4	37,625	6.5
40–49	9885	10.0	524,480	22.1	9885	10.1	59,160	10.3
50–59	16,055	16.3	453,170	19.1	16,045	16.4	96,185	16.7
60–69	17,000	17.3	224,400	9.4	16,975	17.3	101,470	17.6
70–79	20,625	21.0	100,740	4.2	20,545	21.0	121,855	21.1
80+	25,680	26.1	44,115	1.9	25,265	25.8	143,075	24.8
**Practice region**								
East	20,270	20.6	528,820	22.3	20,130	20.6	118,310	20.5
East Midlands	19,140	19.4	435,115	18.3	19,070	19.5	111,940	19.4
London	6910	7.0	125,925	5.3	6855	7.0	40,425	7.0
North East	6350	6.5	137,785	5.8	6345	6.5	37,805	6.6
North West	10,960	11.1	254,725	10.7	10,910	11.1	64,455	11.2
South East	5380	5.5	137,805	5.8	5315	5.4	31,050	5.4
South West	7175	7.3	266,175	11.2	7105	7.3	41,305	7.2
West Midlands	5915	6.0	106,190	4.5	5835	6.0	33,735	5.9
Yorkshire and Humber	16,330	16.6	382,310	16.1	16,315	16.7	97,615	16.9

	Study 2: death							
	Before matching				Matched			
	Case		Control		Case		Control	
	*n*^a^	%	*n*^a^	%	*n*^a^	%	*n*^a^	%
**Number of patients**	22,660	23.0	75,770	77.0	22,330	14.9	127,220	85.1
**Inclusion time**^b^								
Wave 1	6240	27.5	14,055	18.5	6160	27.6	34,895	27.4
Wave 2	12,350	54.5	38,030	50.2	12,195	54.6	70,505	55.4
Wave 3	4070	18.0	23,685	31.3	3975	17.8	21,820	17.2
**Sex**								
Male	13,680	60.4	40,920	54.0	13,500	60.5	77,270	60.7
Female	8980	39.6	34,850	46.0	8830	39.5	49,945	39.3
**Mean age (SD)**	77.2 (12.2)		62.3 (17.6)		77.10 (12.2)		76.5 (11.9)	
**Age group**								
18–29	40	0.2	2845	3.8	35	0.2	160	0.1
30–39	155	0.7	6135	8.1	150	0.7	875	0.7
40–49	450	2.0	9440	12.5	445	2.0	2565	2.0
50–59	1440	6.4	14,615	19.3	1435	6.4	8585	6.7
60–69	3145	13.9	13,855	18.3	3120	14.0	18,290	14.4
70–79	6285	27.7	14,345	18.9	6250	28.0	37,200	29.2
80+	11,150	49.2	14,530	19.2	10,890	48.8	59,545	46.8
**Practice region**								
East	5275	23.3	14,995	19.8	5215	23.4	29,950	23.5
East Midlands	4440	19.6	14,695	19.4	4400	19.7	25,095	19.7
London	1340	5.9	5570	7.4	1325	5.9	7580	6.0
North East	1390	6.1	4960	6.5	1375	6.2	8050	6.3
North West	2530	11.2	8430	11.1	2495	11.2	14,190	11.2
South East	1115	4.9	4260	5.6	1080	4.8	5985	4.7
South West	1495	6.6	5675	7.5	1435	6.4	7500	5.9
West Midlands	1350	6.0	4565	6.0	1300	5.8	7005	5.5
Yorkshire and Humber	3715	16.4	12,615	16.7	3705	16.6	21,865	17.2

a The counts of patients were round to nearest 5 number in line with disclosure controls.

b Wave 1 (early pandemic and first national lockdown, limited COVID-19 test availability for healthcare workers and severe symptoms patients): February–August, 2020; Wave 2 (second national lockdown, COVID-19 availability for wider population): September 2020–April 2021; Wave 3 (end of national lockdown): May–December, 2022.

### Exposures

To measure the long-term impact of repeated antibiotic use on COVID-19 infection outcomes, the maximum exposure time frame was set at 3 years. Since acute effects of antibiotics were not of interest in this study, prescriptions issued in the 6 weeks before the index date were excluded (Fig. 1A). Antibiotics in this study were systemic antibiotics for common infections listed in the British National Formulary (BNF) chapter 5.1 (Antibacterial Drugs), except for BNF5.1.9 (Antituberculosis drugs) and BNF5.1.10 (Antileprotic drugs). A total of 55 unique antibiotic (by molecular structure) was prescribed to this study population. These different antibiotic names were referred to as antibiotic types hereafter to indicate the variety of antibiotics use. Quintile groups based on the number of prior antibiotic prescriptions were created to indicate the frequency of prior antibiotic exposure, where the 1st quintile represents low-frequency users and the 5th quintile high-frequency users. Each quintile was further divided into 1–3 groups based on the number of different antibiotic types a patient was prescribed (i.e., group 1, all antibiotics were the same type; group 3, the patient received a total of three different antibiotic types). Patients without antibiotic use in the prior 3 years were classified as an individual group for both variables.

### Confounding

Demographic variables including patient-level index of multiple deprivation (IMD) quintile (1–5), ethnicity (White, mixed, south Asian, Black, other), smoking status (current, former, never), and care home residence (yes, no) were extracted from the most recent records. Variables of health status were measured in the most recent 5 years, including body mass index (BMI) categorised into underweight, healthy weight, overweight, obese, and a weighted Charlson Comorbidity Index (CCI) group (no comorbidities, low, medium, high, very high).^17^ COVID-19 vaccine or influenza vaccine were recorded within 1 year before index date. Detailed classification criteria are listed in Table 2.

**Table 2:** Baseline characteristics for cases and controls stratified by outcome.

	Study 1: admitted to hospitals				Study 2: death			
	Case		Control		Case		Control	
	n^a^ = 97,880		n^a^ = 576,640		n^a^ = 22,330		n^a^ = 127,220	
	*n*^a^	%	*n*^a^	%	*n*^a^	%	*n*^a^	%
**Ethnicity**								
White	76,040	77.7	404,850	70.2	18,570	83.2	107,545	84.5
South Asian	9895	10.1	44,275	7.7	1820	8.2	8720	6.9
Black	3090	3.2	7535	1.3	510	2.3	2650	2.1
Mixed	1045	1.1	3910	0.7	145	0.6	940	0.7
Other	2670	2.7	8445	1.5	345	1.5	2385	1.9
Unknown	5140	5.3	107,620	18.7	940	4.2	4975	3.9
**BMI category**^b^								
Healthy weight (<18.5 kg/m^2^)	15,590	15.9	117,195	20.3	4720	21.1	25,690	20.2
Underweight (18.5–24.9 kg/m^2^)	1490	1.5	8115	1.4	560	2.5	2255	1.8
Overweight (25–29.9 kg/m^2^)	24,030	24.6	160,760	27.9	5940	26.6	36,675	28.8
Obese (≥30 kg/m^2^)	35,655	36.4	144,830	25.1	7230	32.4	41,340	32.5
Unknown	21,115	21.6	145,740	25.3	3875	17.4	21,265	16.7
**CCI group**^c^								
No comorbidities (0)	41,975	42.9	336,100	58.3	6060	27.1	42,930	33.7
Low (1–2)	45,315	46.3	208,655	36.2	12,210	54.7	66,440	52.2
Medium (3–4)	9515	9.7	29,340	5.1	3595	16.1	15,990	12.6
High (5–6)	1030	1.1	2350	0.4	435	1.9	1800	1.4
Very high (≥7)	45	<0.1	190	<0.1	25	0.1	65	0.1
**Smoking status**^d^								
Never	38,095	38.9	246,155	42.7	6635	29.7	42,105	33.1
Current	7395	7.6	51,305	8.9	1510	6.8	7805	6.1
Former	51,695	52.8	275,020	47.7	14,130	63.3	76,975	60.5
Unknown	690	0.7	4155	0.7	55	0.2	335	0.3
**IMD**^e^								
1 (least deprived)	13,125	13.4	96,390	16.7	3135	14.0	19,180	15.1
2	16,005	16.4	108,345	18.8	3790	17.0	22,700	17.8
3	19,015	19.4	118,705	20.6	4360	19.5	26,210	20.6
4	21,270	21.7	117,735	20.4	4855	21.8	26,215	20.6
5 (most deprived)	26,655	27.2	121,510	21.1	5765	25.8	30,580	24.0
Unknown	1805	1.8	13,950	2.4	415	1.9	2330	1.8
**Care home residents**	3100	3.2	37,315	6.5	1565	7.0	4630	3.6
**COVID-19 vaccine**^f^	22,270	22.8	193,430	33.5	4325	19.4	26,120	20.5
**Flu vaccine**^g^	54,925	56.1	329,720	57.2	15,845	71.0	89,650	70.5

a The counts of patients were round to nearest 5 number in line with disclosure controls.

b BMI, Body Mass Index as recorded within previous 5 years.

c CCI, Charlson Comorbidities Index, measured from 17 weighted conditions, including Myocardial infarction, Congestive heart failure, Peripheral vascular disease, Cerebrovascular disease, Dementia, Chronic pulmonary disease, Connective tissue disease, Ulcer disease, Mild liver disease, Diabetes, Hemiplegia, Moderate or severe renal disease, Diabetes with complications, Any malignancy (including leukaemia and lymphoma), Moderate or severe liver disease, Metastatic solid tumour, AIDS.

d Smoking status and care home residents identified from the most recent clinical records.

e IMD (Index of Multiple Deprivation) quintile measured from patient-level address.

f COVID-19 vaccine identified since vaccination programme started.

g Influenza vaccine identified in previous 1 years.

### Statistical analysis

Descriptive statistics were used to summarise the characteristics of matching variables for the study population, as well as the distribution of confounders for cases and controls at baseline. Conditional logistic regression was conducted to compare the frequency of antibiotic exposure between cases and controls (using R package survival v3.2-3).^18^ Odds ratios (ORs) with 95% confidence intervals (95% CI) were estimated based on unadjusted (crude) and fully adjusted models. Models were adjusted for all confounders listed above. Models included a missingness indicator for ethnicity, BMI, IMD, and smoking status; these are less likely to be less recorded for healthy people so did not meet the “missing at random” assumption for imputation.^19^

### Sensitivity analysis

To check for survival bias, selection criteria for COVID-19 hospital admission and death were tested by using a looser definition that either (1) included COVID-19 as a secondary diagnosis of hospital admission and secondary cause of death or (2) extended the assessment time of COVID-19 outcome from 1 month to 3 months. To investigate potential effect of the definition of severe COVID-19 outcomes, further analyses were done using broader criteria for identifying patients: (1) including both COVID-19 hospital admissions and deaths for study 1 and (2) including both Intensive Care Units (ICU) admissions and COVID-19 deaths for study 2. Furthermore, complete case analysis was used to diagnose the consequence of missing data for ethnicity, BMI, smoking, and IMD. More sensitivity analyses were done to investigate potential confounding effects, including analyses stratifying by age group and sex, removing outliers of total antibiotics consumed (90th, 99th and 99.9th percentiles), adjusting for timing of latest antibiotic prescriptions (in days), as well as 6-week exclusion period of antibiotics. Further conditional logistic regression analysis stratified by age and sex, adjusted for individual diseases, and descriptive statistics for comparing matched and non-matched cases were conducted (See Supplementary Tables S2–S9 and Supplementary Figures S2–S8).

### Software and reproducibility

Data management was performed using Python 3.8.2, with analysis carried out using R 4.0.2. Code for data management and analysis, as well as code lists (Supplementary Table S10), are archived online (https://github.com/opensafely/amr-uom-brit).

### Role of the funding source

The funders of the study had no role in study design, data collection, data analysis, data interpretation, or writing of the report.

## Results

### Study participants

Overall, 2.47 million incident COVID-19 patients were identified before matching between February 1, 2020, and December 31, 2021. Of those, 98,420 (4%) were hospitalised for COVID-19. Among all COVID-19 hospitalised patients, 22,660 (23%) died in 30 days after hospital admission (for study 2). A total of 0.67 million patients was included after matching with replacement. Table 1 shows patient characteristics after matching. Compared with controls, cases were less healthy including higher BMI, CCI, and smoking percentage, more likely to be deprived and less likely to be care home residents (as shown in Table 2). To compare the difference between case and control within matched strata, we randomly picked one control case from multiple matches and found similar results (Supplementary Table S1).

### Antibiotic counts and COVID-19 outcome

Cases had more frequent antibiotic exposure in the prior 3 years compared to controls in both studies (Table 3). The case group had higher odds of receiving antibiotics than controls and the risk rose along with increased exposure quintiles in both crude and adjusted models. For the highest antibiotic exposure quintile, the adjusted OR was 1.80 (95% CI 1.75–1.84) for hospital admissions, and 1.34 (95% CI 1.28–1.41) for death, compared with patients without antibiotic exposure (reference category). Sensitivity analysis stratified by sex and age groups found no distinct change but adjusted OR were slightly higher in 40–59 age group (OR 2.59, 95% CI 2.44–2.75 for study 1; OR 2.26, 95% CI 1.87–2.74 for study 2) (Supplementary Table S2). To investigate the effect of recent antibiotic exposure time, models were adjusted by the timing of the latest antibiotic prescription, though little difference was found (Supplementary Table S3 & Supplementary Figure S2).

**Table 3:** Conditional logistic regression analysis of prior antibiotic exposure levels and COVID-19 outcomes.

Antibiotic quintile	Study 1: admitted to hospitals									
	Case			Control			Crude model		Adjusted model^c^	
	*Med (25*th*, 75*th*)*^a^	*n*^b^	%	*Med (25*th*, 75*th*)*^a^	*n*^b^	%	OR	95% CI	OR	95% CI
No antibiotics	0 (0, 0)	31,830	32.5	0 (0, 0)	237,815	41.2	*ref*		*ref*	
1	1 (1, 1)	16,095	16.4	1 (1, 1)	105,505	18.3	1.17	1.14–1.19	1.03	1.01–1.05
2	2 (2, 2)	10,875	11.1	2 (2, 2)	64,515	11.2	1.32	1.29–1.35	1.10	1.07–1.13
3	3 (3, 3)	7590	7.8	3 (3, 3)	40,695	7.1	1.48	1.44–1.52	1.19	1.15–1.22
4	5 (4, 6)	15,450	15.8	5 (4, 6)	73,125	12.7	1.70	1.66–1.74	1.32	1.29–1.35
5 (most frequent)	14 (10, 23)	16,035	16.4	12 (9, 19)	54,985	9.5	2.41	2.36–2.47	1.80	1.75–1.84

Antibiotic quintile	Study 2: death									
	Case			Control			Crude model		Adjusted model^c^	
	*Med (25*th*, 75*th*)*^a^	*n*^b^	%	*Med (25*th*, 75*th*)*^a^	*n*^b^	%	OR	95% CI	OR	95% CI
No antibiotics	0 (0, 0)	10,845	48.6	0 (0, 0)	67,925	53.4	*ref*		*ref*	
1	1 (1, 1)	3695	16.5	1 (1, 1)	22,060	17.3	1.05	1.01–1.10	1.01	0.97–1.06
2	2 (2, 2)	1980	8.9	2 (2, 2)	10,875	8.5	1.15	1.09–1.21	1.07	1.02–1.13
3	3 (3, 3)	1305	5.8	3 (3, 3)	6315	5.0	1.30	1.22–1.39	1.21	1.13–1.29
4	5 (4, 5)	1830	8.2	5 (4, 5)	8625	6.8	1.35	1.28–1.42	1.25	1.18–1.32
5 (most frequent)	13 (9, 28)	2680	12.0	13 (9, 24)	11,415	9.0	1.48	1.41–1.55	1.34	1.28–1.41

a Median (25th precentile, 75th percentile) of number of antibiotic prescriptions in each quintile group.

b The counts of patients were round to nearest 5 number in line with disclosure controls.

c Adjusted for ethnicity, BMI category, CCI group, smoking status, IMD, care home residents, COVID-19 and influenza vaccine.

### Antibiotic variety and COVID-19 outcome

**Fig. 2:**
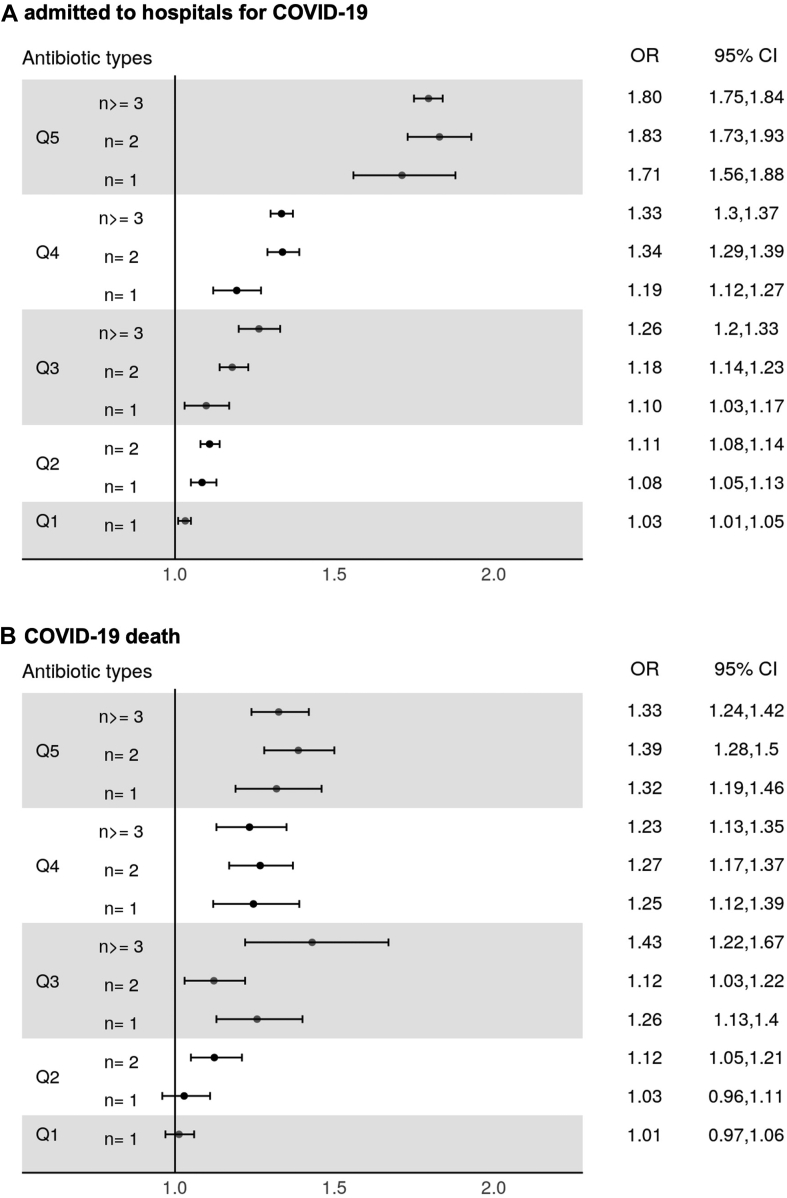
**Adjusted ORs for COVID-19 outcomes (A. admitted to hospitals; B. death) stratified by number of antibiotic types in the 3 years by quintile (Q1–Q5) of total number of prior antibiotic prescription.**

To understand the impact of antibiotics variety on COVID-19 outcome, the breakdown of antibiotic type for each frequency quintile was divided by 1, 2, and 3+ types. Fig. 2 shows that cases were at a higher probability to be exposed to diverse antibiotics and this association increased with the frequency of antibiotic exposure. In study 1, the adjusted OR of quintile 5 exposure (the most frequent) with more than 3 antibiotic types was around 2 times larger than quintile 1 exposure with only 1 type (OR 1.80, 95% CI 1.75–1.84 vs. OR 1.03, 95% CI 1.01–1.05). In study 2, a similar exposure-response association was observed although the magnitude of ORs was smaller. The highest OR was for quintile 3 (middle quintile antibiotic use) with 3 or more types (OR 1.43, 95% CI 1.22–1.67) where no significant difference was found in quintile 1 and 2 with only 1 type. Sensitivity analysis was used to detect the possible outliers of antibiotics prescriptions. After removing patients with the 99.99th, 99th, and 90th percentile of total prescriptions, the overall trend of OR still increased with quintiles and number of types (Supplementary Figure S3), except for quintile 5 with number of types greater than 3 in study 1, where excluding 90th percentile outliers reduced the odds to 1.48 (95% CI 1.42–1.53).

### Additional analysis for exclusion period

The main analysis excluded antibiotics in the previous 6 weeks to investigate the long-term effects of varying antibiotic exposure frequency. However, considering that there might be potential confounding from recent antibiotic use, an additional analysis was conducted to adjust for history of antibiotic use during the exclusion period. Three different approaches were applied to measure the confounders including antibiotic exposure (yes/no), the number of antibiotic prescriptions and the number of antibiotic types from 6 weeks before the index date. No obvious change was observed for severe outcome in study 2 but overall adjusted OR decreased in study 1. For example, after adjusting counts of antibiotics in recent 6 weeks and all confounders, OR of quintile 5 decreased to 1.33 (95% CI 1.30–1.37) (Supplementary Table S4) and OR of more than 3 types in quintile 5 decreased to 1.33 (95% 1.29–1.37) (Supplementary Figure S4).

### Sensitivity analysis for study definition

The results did not change by relaxing the definition of COVID-19 hospital admissions and COVID-19 deaths as secondary diagnosis inclusive or extending the time frame for same COVID-19 infection episode (Supplementary Table S5 & Supplementary Figure S5). Results were similar when applying broader definition of severe outcome (adding COVID-19 deaths for study 1). However, including ICU admission as severe outcome for study 2 reduced the OR (Supplementary Table S6 & Supplementary Figure S6).

### Sensitivity analysis for comorbidities

The prevalence difference of 17 different comorbidities between case and control groups ranged from <1% to 8.2% (Supplementary Table S7A), which showed no major difference in distribution. To understand whether adjustment for a weighted CCI affected the results, an additional model adjusted for 17 individual diseases. This found similar results to the main analysis (Supplementary Table S7B & Supplementary Figure S7).

### Sensitivity analysis for exclusion and missing data

Though implementing matching with replacement improved the matching performance, less than 1% cases still failed to find matched controls due to very old ages (Supplementary Table S8 & Supplementary Figure S8). Around 20% of BMI were missing in our analysis. A complete case sensitivity analysis was performed. The adjusted OR of antibiotic exposure frequency only changed minimally compared the original models which included missing data in both studies (Supplementary Table S9 & Supplementary Figure S9).

## Discussion

We found an association between prior antibiotic exposure frequency and severe COVID-19 outcomes with a dose–response. This finding was consistent with a previous Spanish study. However, that study used a simpler measure for antibiotic exposure and only covered the first 4 months of the pandemic.^20^ To our knowledge, the present study is the first to explore the relationship between repeated prior 3-year antibiotics exposure and subsequent severity of COVID-19 infections covering most of the pandemic. Additionally, an association was observed between antibiotic diversity (number of different prior antibiotic types) and COVID-19 related hospital admission.

There may be several explanations for our findings, ranging from a direct effect of antibiotics to potential confounding. A direct effect of antibiotics could involve disruption of the gut microbiome. According to a recent review, the structure of the gut microbiome is relatively stable in adulthood. However, antibiotics are thought to cause acute gut dysbiosis and affect resilience to perturbation due to reduced diversity or richness of bacteria. The gut microbiota play an important role in regulating human immune and metabolic systems, thus frequent antibiotic perturbation might result in adverse effects.^21^ For example, Sultan (2019) discovered in a population-based database an association between frequency of antibiotic exposure and risk of rheumatoid arthritis (an autoimmune disease).^9^ Alternatively, antibiotics might also impact the gut resistome which comprises of antibiotic resistance genes (ARG) in gut flora and thus lead to antibiotic resistance, increasing COVID-19 patients' susceptibility to secondary bacterial infection and difficulty in treatment. A previous observational study found that patients with frequent intermittent antibiotic exposure were more likely to suffer from infection-related complications.^8^ A meta-analysis also reported that 24% of COVID-19 hospital patients presented bacterial resistant co-infection.^22^ Furthermore, a number of studies have analysed faecal samples and found that the composition of gut microbiota in particular, bacterial species for potential immunomodulation were depleted in COVID-19 patients.^23^^,^^24^ The study by Yeoh et al. (2021) evaluated stool samples and found that gut microbiome composition was significantly altered in patients with COVID-19 compared with non-COVID-19 individuals, which was also correlated with the increased levels of cytokines and inflammatory markers in patients with COVID-19.^24^ In addition, a recent study found that COVID-19 patients with empiric antibiotic treatment presented with an abundance of ARGs compared to healthy controls.^25^ Since the samples were collected during COVID-19 hospital admission, a temporal relationship between antibiotics and human health cannot be determined, but these findings support the hypothesis that antibiotics could adversely impact human health through disruption of gut microbiota.

The association observed in this study might be subject to confounding as patients using antibiotics repeatedly may have been more immunocompromised, increasing susceptibility to infections and adverse clinical outcomes. A South Korean study estimated that around 13.5% of COVID-19 patients had history of malignancy, HIV/AIDS, organ transplantation or prescriptions for immunosuppressants.^26^ However, our sensitivity analysis adjusting for 17 individual diseases including HIV/AIDS and malignancy showed that the ORs did not change substantially. A future study may attempt to elaborate immunosuppressing conditions among patients exposed to antibiotics. However, the high prevalence of repeated antibiotic exposure in patients in primary care may indicate that confounding by being immunocompromised may not fully explain the current findings. Importantly, there is little evidence to suggest that repeated intermittent antibiotic exposure is actually effective in reducing infection-related complications.^8^ A review by Costelloe et al. (2010) reported that individuals prescribed an antibiotic in primary care for a respiratory or urinary infection are more likely to develop bacterial resistance to that antibiotic.^27^

The strength of this study was the ability to look at longer-term antibiotic prescribing using OpenSAFELY that linked multiple data sources. It covered a population of more than 20 million patients in England with comprehensive health care data provided. This study has several limitations. First, there might be potential misclassification of cases and controls in this study for several reasons. A COVID-19 episode was defined as lasting one month in line with previous research suggesting the natural trajectory of a COVID-19 infection.^28^^,^^29^ Besides, a sensitivity analysis considering COVID-19 infections lasting for 3 months found no difference. Further analyses by relaxing the criteria of selecting cases with severe outcome also presented similar results, suggesting that prior antibiotic exposure had effect on the severe COVID-19 outcomes regardless of using strict or loose case definitions. Secondly, as we could not fully match the oldest cases (<1%), the results from this study cannot be inferred to this population. Finally, despite that current data had data missing BMI, ethnicity, smoking, and IMD, a complete case analysis found consistent results.

As a trial randomising patients to different antibiotic histories cannot be conducted, the interpretation of this observational study findings may need to consider wider evidence of benefits as well as adverse effects of repeated intermittent antibiotic exposure. There is little evidence to suggest that repeated intermittent antibiotic exposure is effective in reducing infection-related complications. On the other hand, there is accumulating evidence that this antibiotic use may be ineffective or even unsafe, particularly when other research has found that gut microbiome composition was significantly altered in patients with COVID-19, this may explain the accumulative effect of prior antibiotic exposure on covid-19 infection severity observed in this study. A recent commentary around repeated antibiotic prescribing highlighted the need to promote behavioural change. Antibiotic stewardship strategies to reduce inappropriate repeat antibiotic prescribing could include review of these patients with specialised toolkits and habits of collegial reviews.^30^ Additional strategies could include to clearly outline in antibiotic prescribing guidelines what type of antibiotic is unlikely to be of value.^31^ Currently, the common infection guidelines in England (as developed by the National Institute for Health and Care Excellence) do not provide guidance around commonly encountered challenges such as the high levels of antibiotic treatment failure, repeated antibiotic use and a patient's risk of developing resistance to the antibiotic.^32^^,^^33^ Furthermore, shared decision-making between patients and clinicians could also include a balanced discussion of the risks for a patient of repeated antibiotic use and future resistance.^31^

To conclude, we found that antibiotic exposure frequency and diversity were associated with COVID-19 clinical outcome severity. Given the known effects of antibiotics on the gut microbiome, it seems advisable to discourage the regular practice of indiscriminately prescribing antibiotics repeatedly and intermittently given their uncertain benefits and likely risks.

## Contributors

All authors contributed to and approved the final manuscript. Design of study, TVS, VP, DW, YY. Analysis: YY. Interpretation of results and first draft of manuscript: YY, VP, DW, TvS. YT, XZ, AF, VP, and JM accessed and verified the underlying data. TvS is the guarantor for the article, and accept full responsibility for the work and/or the conduct of the study, had access to the data, and controlled the decision to publish. The corresponding author attests that all listed authors meet authorship criteria and that no others meeting the criteria have been omitted.

## Data sharing statement

All data were linked, stored and analysed securely within the OpenSAFELY platform https://opensafely.org/. Data include pseudonymised data such as coded diagnoses, medications and physiological parameters. No free text data are included. All code is shared openly for review and re-use under MIT open license (https://github.com/opensafely/amr-uom-brit/). Detailed pseudonymised patient data is potentially re-identifiable and therefore not shared. We rapidly delivered the OpenSAFELY data analysis platform without prior funding to deliver timely analyses on urgent research questions in the context of the global COVID-19 health emergency: now that the platform is established we are developing a formal process for external users to request access in collaboration with NHS England; details of this process will be published shortly on OpenSAFELY.org.

Access to the underlying identifiable and potentially re-identifiable pseudonymised electronic health record data is tightly governed by various legislative and regulatory frameworks, and restricted by best practice. The data in OpenSAFELY is drawn from General Practice data across England where TPP is the Data Processor. TPP developers (CB, JP, FH, SH, JC) initiate an automated process to create pseudonymised records in the core OpenSAFELY database, which are copies of key structured data tables in the identifiable records. These are linked onto key external data resources that have also been pseudonymised via SHA-512 one-way hashing of NHS numbers using a shared salt. DataLab developers and PIs holding contracts with NHS England have access to the OpenSAFELY pseudonymised data tables as needed to develop the OpenSAFELY tools. These tools in turn enable researchers with OpenSAFELY Data Access Agreements to write and execute code for data management and data analysis without direct access to the underlying raw pseudonymised patient data, and to review the outputs of this code. All code for the full data management pipeline—from raw data to completed results for this analysis—and for the OpenSAFELY platform as a whole is available for review at github.com/OpenSAFELY. The data management and analysis code for this paper was led by YY and contributed to by JM, VP, XZ and AF.

## Declaration of interests

All authors declare the following: BG and OpenSAFELY has received research funding from the Laura and John Arnold Foundation, the NHS National Institute for Health Research (NIHR), the NIHR School of Primary Care Research, NHS England, the NIHR Oxford Biomedical Research Centre, the Mohn-Westlake Foundation, NIHR Applied Research Collaboration Oxford and Thames Valley, the Wellcome Trust, the Good Thinking Foundation, Health Data Research UK, the Health Foundation, the World Health Organisation, UKRI MRC, Asthma UK, the British Lung Foundation, and the Longitudinal Health and Wellbeing strand of the National Core Studies programme; he is a Non-Executive Director at NHS Digital; he also receives personal income from speaking and writing for lay audiences on the misuse of science. AM has received consultancy fees (from https://inductionhealthcare.com) and is member of RCGP health informatics group and the NHS Digital GP data Professional Advisory Group that advises on access to GP Data for Pandemic Planning and Research (GDPPR). For the latter, he received payment for the GDPPR role.

## Appendix A Supplementary data

Supplementary Figures S1–S9, Tables S1–S10 and Protocol
