# Supplementary material for: Repeated antibiotic exposure and risk of hospitalisation and death following COVID-19 infection (OpenSAFELY): a matched case–control study
Source: eClinicalMedicine. 2023 Jul 5;61:102064. doi: 10.1016/j.eclinm.2023.102064 (PMC10388579; doi:10.1016/j.eclinm.2023.102064)
Supplement: Supplementary Figures S1–S9, Tables S1–S10 and Protocol [file mmc1.docx]

Contents

[Supplementary Tables 2](#_Toc136329724)

[Supplementary Table 1. Characteristics of study cohorts after matching (1 control randomly picked) 2](#_Toc136329725)

[Supplementary Table 2A. Analysis stratified by sex 4](#_Toc136329726)

[Supplementary Table 2B. Analysis stratified by age groups 5](#_Toc136329727)

[Supplementary Table 3. Analysis adjusted for timing of antibiotics prescribed 6](#_Toc136329728)

[Supplementary Table 4. Analysis adjusted for antibiotics in recent 6 weeks 7](#_Toc136329729)

[Supplementary Table 5. Analysis included secondary Covid-19 outcomes & prolonged duration (90days) of same Covid-19 infection 8](#_Toc136329730)

[Supplementary Table 6. Analysis using different definition of severe outcome 9](#_Toc136329731)

[Supplementary Table 7A. Distribution of individual disease 10](#_Toc136329732)

[Supplementary Table 7B. Analysis adjusted for individual disease 11](#_Toc136329733)

[Supplementary Table 8. Comparison of matched and non-matched cases 12](#_Toc136329734)

[Supplementary Table 9. Complete case analysis 13](#_Toc136329735)

[Supplementary Table 10. Code lists used for variable definition 14](#_Toc136329736)

[Supplementary Figures 16](#_Toc136329737)

[Supplementary Figure 1. Patient selection criteria and variable measurement 16](#_Toc136329738)

[Supplementary Figure 2. Analysis adjusted for timing of antibiotics prescribed 19](#_Toc136329739)

[Supplementary Figure 3. Analysis removing outliers 21](#_Toc136329740)

[Supplementary Figure 4. Analysis adjusted for antibiotics in recent 6 weeks 24](#_Toc136329741)

[Supplementary Figure 5. Analysis included secondary Covid-19 outcomes & prolonged duration (90days) of same Covid-19 infection 27](#_Toc136329742)

[Supplementary Figure 6. Analysis using different definition of severe outcome 29](#_Toc136329743)

[Supplementary Figure 7. Analysis adjusted for individual disease 30](#_Toc136329744)

[Supplementary Figure 8. Age distribution of matched and non-matched cases 31](#_Toc136329745)

[Supplementary Figure 9. Complete case analysis 32](#_Toc136329746)

[Supplementary Protocol 33](#_Toc136329747)

# Supplementary Tables

## **Supplementary Table 1. Characteristics of study cohorts after matching (1 control randomly picked)**

|  | **Study 1: admitted to hospitals** | | | | **Study 2: death** | | | |
| --- | --- | --- | --- | --- | --- | --- | --- | --- |
|  | **case** | | **control** | | **case** | | **control** | |
|  | **n^8^** | **%** | **n^8^** | **%** | **n^8^** | **%** | **n^8^** | **%** |
| **number of patients** | 97,880 |  | 97,880 |  | 22,330 |  | 22,330 |  |
| **Inclusion time^1^** |  |  |  |  |  |  |  |  |
| wave 1 | 19,880 | 20.3 | 19,880 | 20.3 | 6,160 | 27.6 | 6,160 | 27.7 |
| wave 2 | 50,295 | 51.4 | 50,295 | 51.4 | 12,195 | 54.6 | 12,195 | 54.9 |
| wave 3 | 27,705 | 28.3 | 27,705 | 28.3 | 3,975 | 17.8 | 3,975 | 17.9 |
| **Sex** |  |  |  |  |  |  |  |  |
| male | 54,270 | 55.4 | 54,270 | 55.4 | 13,500 | 60.5 | 13,500 | 60.5 |
| **Mean age (SD)** | 65.6 (17.6) | | 65.5 (17.6) | | 77.1 (12.1) | | 76.8 (12.0) | |
| **Age group** |  |  |  |  |  |  |  |  |
| 18-29 | 2,880 | 2.9 | 2,880 | 2.9 | 35 | 0.2 | 30 | 0.1 |
| 30-39 | 6,285 | 6.4 | 6,310 | 6.4 | 150 | 0.7 | 170 | 0.8 |
| 40-49 | 9,885 | 10.1 | 9,895 | 10.1 | 445 | 2.0 | 455 | 2.0 |
| 50-59 | 16,045 | 16.4 | 16,070 | 16.4 | 1,435 | 6.4 | 1,470 | 6.6 |
| 60-69 | 16,975 | 17.3 | 17,055 | 17.4 | 3,120 | 14.0 | 3,150 | 14.1 |
| 70-79 | 20,545 | 21.0 | 20,690 | 21.1 | 6,250 | 28.0 | 6,345 | 28.4 |
| 80+ | 25,265 | 25.8 | 24,980 | 25.5 | 10,890 | 48.8 | 10,705 | 48.0 |
| **Practice region** |  |  |  |  |  |  |  |  |
| East | 20,130 | 20.6 | 20,130 | 20.6 | 5,215 | 23.4 | 5,215 | 23.4 |
| East Midlands | 19,070 | 19.5 | 19,070 | 19.5 | 4,400 | 19.7 | 4,400 | 19.7 |
| London | 6,855 | 7.0 | 6,855 | 7.0 | 1,325 | 5.9 | 1,325 | 5.9 |
| North East | 6,345 | 6.5 | 6,345 | 6.5 | 1,375 | 6.2 | 1,375 | 6.2 |
| North West | 10,910 | 11.1 | 10,910 | 11.1 | 2,495 | 11.2 | 2,495 | 11.2 |
| South East | 5,315 | 5.4 | 5,315 | 5.4 | 1,080 | 4.8 | 1,080 | 4.8 |
| South West | 7,105 | 7.3 | 7,105 | 7.3 | 1,435 | 6.4 | 1,435 | 6.4 |
| West Midlands | 5,835 | 6.0 | 5,835 | 6.0 | 1,300 | 5.8 | 1,300 | 5.8 |
| Yorkshire and Humber | 16,315 | 16.7 | 16,315 | 16.7 | 3,705 | 16.6 | 3,705 | 16.6 |
| **Ethnicity** |  |  |  |  |  |  |  |  |
| White | 76,040 | 77.7 | 69,095 | 70.6 | 18,570 | 83.2 | 18,930 | 84.8 |
| South Asian | 9,895 | 10.1 | 7,535 | 7.7 | 1,820 | 8.2 | 1,505 | 6.7 |
| Black | 3,090 | 3.2 | 1,285 | 1.3 | 510 | 2.3 | 435 | 1.9 |
| Mixed | 1,045 | 1.1 | 690 | 0.7 | 145 | 0.6 | 150 | 0.7 |
| Other | 2,670 | 2.7 | 1,410 | 1.4 | 345 | 1.5 | 400 | 1.8 |
| Unknown | 5,140 | 5.3 | 17,860 | 18.2 | 940 | 4.2 | 910 | 4.1 |
| **BMI category^2^** |  |  |  |  |  |  |  |  |
| Healthy weight (<18.5 kg/m^2^) | 15,590 | 15.9 | 20,135 | 20.6 | 4,720 | 21.1 | 4,635 | 20.8 |
| Underweight (18.5-24.9 kg/m^2^) | 1,490 | 1.5 | 1,405 | 1.4 | 560 | 2.5 | 425 | 1.9 |
| Overweight (25-29.9 kg/m^2^) | 24,030 | 24.6 | 27,450 | 28.0 | 5,940 | 26.6 | 6,480 | 29.0 |
| Obese (≥30 kg/m^2^) | 35,655 | 36.4 | 24,415 | 24.9 | 7,230 | 32.4 | 7,090 | 31.8 |
| Unknown | 21,115 | 21.6 | 24,470 | 25.0 | 3,875 | 17.4 | 3,700 | 16.6 |
| **CCI group^3^** |  |  |  |  |  |  |  |  |
| No comorbidities (0) | 41,975 | 42.9 | 56,515 | 57.7 | 6,060 | 27.1 | 7,550 | 33.8 |
| Low (1-2) | 45,315 | 46.3 | 35,815 | 36.6 | 12,210 | 54.7 | 11,725 | 52.5 |
| Medium (3-4) | 9,515 | 9.7 | 5,080 | 5.2 | 3,595 | 16.1 | 2,740 | 12.3 |
| High (5-6) | 1,030 | 1.1 | 440 | 0.4 | 435 | 1.9 | 300 | 1.3 |
| Very high (≥ 7) | 45 | <0.1 | 35 | <0.1 | 25 | 0.1 | 10 | <0.1 |
| **Smoking status^4^** |  |  |  |  |  |  |  |  |
| Never | 38,095 | 38.9 | 41,735 | 42.6 | 6,635 | 29.7 | 7,375 | 33.0 |
| Current | 7,395 | 7.6 | 8,520 | 8.7 | 1,510 | 6.8 | 1,315 | 5.9 |
| Former | 51,695 | 52.8 | 46,880 | 47.9 | 14,130 | 63.3 | 13,600 | 60.9 |
| Unknown | 690 | 0.7 | 740 | 0.8 | 55 | 0.2 | 40 | 0.2 |
| **IMD^5^** |  |  |  |  |  |  |  |  |
| 1﻿(least deprived) | 13,125 | 13.4 | 16,565 | 16.9 | 3,135 | 15.2 | 3,405 | 15.2 |
| 2 | 16,005 | 16.4 | 18,430 | 18.8 | 3,790 | 17.9 | 3,995 | 17.9 |
| 3 | 19,015 | 19.4 | 20,180 | 20.6 | 4,360 | 19.5 | 4,660 | 20.9 |
| 4 | 21,270 | 21.7 | 19,950 | 20.4 | 4,855 | 21.8 | 4,595 | 20.6 |
| 5(most deprived) | 26,655 | 27.2 | 20,495 | 20.9 | 5,765 | 25.8 | 5,250 | 23.5 |
| Unknown | 1,805 | 1.8 | 2,265 | 2.3 | 415 | 1.9 | 425 | 1.9 |
| **Care home residents** | 3,100 | 3.2 | 6,385 | 6.5 | 1,565 | 7.0 | 845 | 3.8 |
| **Covid-19 vaccine^6^** | 22,270 | 22.8 | 32,615 | 33.3 | 4,325 | 19.4 | 4,710 | 21.1 |
| **Flu vaccine^7^** | 54,925 | 56.1 | 56,200 | 57.4 | 15,845 | 70.9 | 15,615 | 69.9 |
| 1. Wave 1(early pandemic and first national lockdown, limited COVID-19 test availability for healthcare workers and severe symptoms patients): February to August, 2020; Wave 2(second national lockdown, COVID-19 availability for wider population): September 2020 to April 2021; Wave3(end of national lockdown): May to December, 2022 2. BMI, Body Mass Index in recent 5 years 3. CCI, Charlson Comorbidities Index, measured from 17 weighted conditions, including Myocardial infarct, Congestive heart failure, Peripheral vascular disease, Cerebrovascular disease, Dementia, Chronic pulmonary disease, Connective tissue disease, Ulcer disease, Mild liver disease, Diabetes, Hemiplegia, Moderate or severe renal disease, diabetes with complications, Any malignancy(including leukemia and lymphoma), Moderate or severe liver disease, Metastatic solid tumor, AIDS 4. smoking status and care home residents identified from the most recent clinical records 5.IMD, Index of Multiple Deprivation, measured form patient-level address  6. Covid-19 vaccine identified since vaccination programme started 7. flu vaccine identified in recent 1 year | | | | | | | | |
| 8. The counts of patients were round to nearest 5 number in line with disclosure controls. | | | | | | | | |

## **Supplementary Table 2A. Analysis stratified by sex**

| **Study 1: admitted to hospitals** | | | | | | |
| --- | --- | --- | --- | --- | --- | --- |
|  | **male** | | | **female** | | |
| **Antibiotic quintile** | **Med (25th, 75th)** | **crude OR** | **adjusted OR** | **Med (25th, 75th)** | **crude OR** | **adjusted OR** |
| no antibiotics | 0(0,0) | ref | ref | 0(0,0) | ref | ref |
| 1 | 1(1,1) | 1.17(1.14-1.20) | 1.03(1.00-1.06) | 1(1,1) | 1.17(1.13-1.21) | 1.04(1.01-1.08) |
| 2 | 2(2,2) | 1.31(1.27-1.35) | 1.10(1.06-1.13) | 2(2,2) | 1.34(1.29-1.39) | 1.11(1.07-1.15) |
| 3 | 3(3,3) | 1.46(1.41-1.52) | 1.19(1.15-1.24) | 3(3,3) | 1.51(1.45-1.57) | 1.19(1.14-1.24) |
| 4 | 5(4,6) | 1.68(1.63-1.73) | 1.31(1.27-1.36) | 5(4,6) | 1.73(1.68-1.79) | 1.34(1.29-1.38) |
| 5 (most frequent) | 12(9,20) | 2.28(2.21-2.36) | 1.75(1.69-1.81) | 12(9,20) | 2.55(2.47-2.63) | 1.83(1.76-1.89) |
| **Study 2: death** | | | | | | |
|  | **male** | | | **female** | | |
| **Antibiotic quintile** | **Med (25th, 75th)** | **crude OR** | **adjusted OR** | **Med (25th, 75th)** | **crude OR** | **adjusted OR** |
| no antibiotics | 0(0,0) | ref | ref | 0(0,0) | ref | ref |
| 1 | 1(1,1) | 1.08(1.02-1.14) | 1.04(0.99-1.10) | 1(1,1) | 1.01(0.95-1.08) | 0.97(0.91-1.04) |
| 2 | 2(2,2) | 1.14(1.06-1.22) | 1.06(0.99-1.14) | 2(2,2) | 1.15(1.06-1.25) | 1.09(1.00-1.18) |
| 3 | 3(3,3) | 1.30(1.20-1.42) | 1.20(1.10-1.31) | 3(3,3) | 1.30(1.18-1.43) | 1.22(1.11-1.34) |
| 4 | 5(4,5) | 1.35(1.25-1.46) | 1.25(1.16-1.35) | 5(4,5) | 1.34(1.23-1.45) | 1.25(1.15-1.35) |
| 5 (most frequent) | 13(9,24) | 1.51(1.41-1.61) | 1.35(1.26-1.45) | 13(9,26) | 1.45(1.35-1.55) | 1.33(1.24-1.43) |
| 1. Med (25th, 75th): median (25th percentile, 75th percentile) of total antibiotics | | | | |  |  |
| 2. adjusted OR: adjusted for ethnicity, BMI category, CCI group, smoking status, IMD, care home residents, Covid-19 and flu vaccine | | | | | | |

## **Supplementary Table 2B. Analysis stratified by age groups**

| **Study 1: admitted to hospitals** | | | | | | | | | | | |  |
| --- | --- | --- | --- | --- | --- | --- | --- | --- | --- | --- | --- | --- |
|  | **age 18-39** | | **age 40-59** | | | **age 60-79** | | | **age 80+** | | |  |
| **Antibiotic quintile** | **Med (25th, 75th)** | **crude OR (95%CI)** | **Med (25th, 75th)** | **crude OR (95%CI)** | **adjusted OR (95%CI)** | **Med (25th, 75th)** | **crude OR (95%CI)** | **adjusted OR (95%CI)** | **Med (25th, 75th)** | **crude OR (95%CI)** | **adjusted OR (95%CI)** |  |
| no antibiotics | 0(0,0) | ref | 0(0,0) | ref | ref | 0(0,0) | ref | ref | 0(0,0) | ref | ref |  |
| 1 | 1(1,1) | 1.24(1.17-1.32) | 1(1,1) | 1.19(1.15-1.24) | 1.02(0.98-1.06) | 1(1,1) | 1.16(1.12-1.20) | 1.03(1.00-1.07) | 1(1,1) | 1.01(0.96-1.06) | 0.99(0.95-1.04) |  |
| 2 | 2(2,2) | 1.41(1.31-1.52) | 2(2,2) | 1.43(1.37-1.50) | 1.11(1.05-1.16) | 2(2,2) | 1.34(1.29-1.39) | 1.12(1.07-1.16) | 2(2,2) | 1.01(0.96-1.06) | 1.00(0.95-1.05) |  |
| 3 | 3(3,3) | 1.63(1.49-1.78) | 3(3,3) | 1.62(1.53-1.72) | 1.15(1.08-1.22) | 3(3,3) | 1.49(1.42-1.55) | 1.19(1.13-1.25) | 3(3,4) | 1.11(1.06-1.17) | 1.10(1.04-1.16) |  |
| 4 | 5(4,6) | 1.93(1.79-2.08) | 5(4,6) | 2.1(2.00-2.20) | 1.41(1.34-1.48) | 5(4,6) | 1.79(1.73-1.86) | 1.34(1.29-1.39) | 5(4,6) | 1.14(1.09-1.19) | 1.14(1.09-1.19) |  |
| 5 (most frequent) | 10(9,15) | 3.53(3.20-3.90) | 12(9,18) | 4.13(3.92-4.36) | 2.59(2.44-2.75) | 13(10,22) | 2.82(2.72-2.91) | 1.91(1.84-1.98) | 12(9,19) | 1.33(1.27-1.38) | 1.31(1.25-1.36) |  |
| **Study 2: death** | | | | | | | | | | | |  |
|  | **age 18-39** | | **age 40-59** | | | **age 60-79** | | | **age 80+** | | |  |
| **Antibiotic quintile** | **Med (25th, 75th)** | **crude OR (95%CI)** | **Med (25th, 75th)** | **crude OR (95%CI)** | **adjusted OR (95%CI)** | **Med (25th, 75th)** | **crude OR (95%CI)** | **adjusted OR (95%CI)** | **Med (25th, 75th)** | **crude OR (95%CI)** | **adjusted OR (95%CI)** |  |
| no antibiotics | 0(0,0) | ref | 0(0,0) | ref | ref | 0(0,0) | ref | ref | 0(0,0) | ref | ref |  |
| 1 | 1(1,1) | 1.35(0.87-2.10) | 1(1,1) | 1.19(1.03-1.37) | 1.08(0.93-1.25) | 1(1,1) | 1.07(1.00-1.14) | 1.02(0.96-1.09) | 1(1,1) | 1(0.95-1.06) | 0.98(0.93-1.04) |  |
| 2 | 2(2,2) | 0.93(0.42-2.03) | 2(2,2) | 1.38(1.14-1.67) | 1.2(0.98-1.47) | 2(2,2) | 1.18(1.08-1.28) | 1.09(1.00-1.19) | 2(2,2) | 1.08(1.01-1.16) | 1.04(0.96-1.12) |  |
| 3 | 3(3,3) | 1.08(0.47-2.53) | 3(3,3) | 1.72(1.34-2.20) | 1.38(1.06-1.78) | 3(3,3) | 1.31(1.18-1.45) | 1.19(1.07-1.32) | 3(3,3) | 1.24(1.14-1.35) | 1.18(1.08-1.29) |  |
| 4 | 5(4,5) | 5.64(2.69-11.82) | 5(4,5) | 2.03(1.62-2.54) | 1.54(1.22-1.95) | 5(4,5) | 1.34(1.23-1.46) | 1.22(1.12-1.33) | 5(4,5) | 1.26(1.17-1.36) | 1.22(1.13-1.32) |  |
| 5 (most frequent) | 13(8,34) | 5.15(2.74-9.68) | 13(9,23) | 3.03(2.54-3.63) | 2.26(1.87-2.74) | 14(9,26) | 1.69(1.58-1.81) | 1.51(1.41-1.62) | 13(9,24) | 1.16(1.08-1.25) | 1.10(1.02-1.18) |  |
| 1. Med (25th, 75th): median (25th percentile, 75th percentile) of total antibiotics | | | | | |  |  |  |  |  |  |  |
| 2. adjusted OR: adjusted for ethnicity, BMI category, CCI group, smoking status, IMD, care home residents, Covid-19 and flu vaccine; adjusted model for age 18-39 was not converged due to limited patients. | | | | | | | | | | | |  |

## **Supplementary Table 3. Analysis adjusted for timing of antibiotics prescribed**

| **Study 1: admitted to hospitals** | | | | |  |
| --- | --- | --- | --- | --- | --- |
|  | **excluding antibiotics in recent 6 weeks** | | **including antibiotics in recent 6 weeks** | |  |
| **Antibiotic quintile** | **adjusted OR1 (95% CI)** | **adjusted OR2 (95% CI)** | **adjusted OR1 (95% CI)** | **adjusted OR2 (95% CI)** |  |
| no antibiotics | ref | ref | ref | ref |  |
| 1 | 1.19(1.17-1.22) | 1.05(1.03-1.07) | 1.16(1.14-1.19) | 1.03(1.01-1.05) |  |
| 2 | 1.36(1.33-1.40) | 1.13(1.10-1.16) | 1.31(1.28-1.34) | 1.09(1.07-1.12) |  |
| 3 | 1.53(1.49-1.58) | 1.23(1.19-1.26) | 1.46(1.42-1.50) | 1.18(1.14,1.21) |  |
| 4 | 1.77(1.72-1.81) | 1.37(1.34-1.40) | 1.67(1.63-1.70) | 1.30(1.27-1.33) |  |
| 5 (most frequent) | 2.48(2.42-2.54) | 1.84(1.80-1.89) | 2.25(2.20-2.31) | 1.69(1.64-1.73) |  |
| **Study 2: death** | | | | |  |
|  | **excluding antibiotics in recent 6 weeks** | | **including antibiotics in recent 6 weeks** | |  |
| **Antibiotic quintile** | **adjusted OR1 (95% CI)** | **adjusted OR2 (95% CI)** | **Adjusted OR1 (95% CI)** | **adjusted OR2 (95% CI)** |  |
| no antibiotics | ref | ref | ref | ref |  |
| 1 | 1.16(1.10-1.23) | 1.12(1.06-1.19) | 1.05(1.01-1.09) | 1.01(0.97-1.05) |  |
| 2 | 1.24(1.16-1.32) | 1.16(1.09-1.24) | 1.14(1.08-1.20) | 1.07(1.02-1.13) |  |
| 3 | 1.39(1.30-1.49) | 1.29(1.20-1.38) | 1.30(1.22-1.38) | 1.20(1.13-1.28) |  |
| 4 | 1.42(1.34-1.50) | 1.32(1.24-1.40) | 1.34(1.26-1.41) | 1.24(1.17-1.31) |  |
| 5 (most frequent) | 1.52(1.45-1.60) | 1.38(1.31-1.45) | 1.44(1.37-1.52) | 1.31(1.25-1.38) |  |
| 1. OR1: adjusted for the time interval(days) between index date and latest antibiotic prescription 2. OR2: adjusted for the time interval(days) between index date and latest antibiotic prescription, as well as confounders (ethnicity, BMI category, CCI group, smoking status, IMD, care home residents, Covid-19 and flu vaccine) | | | | |  |
|  | | | | |  |

## **Supplementary Table 4. Analysis adjusted for antibiotics in recent 6 weeks**

| **Study 1: admitted to hospitals** | | | | | | | | | | |  |  |
| --- | --- | --- | --- | --- | --- | --- | --- | --- | --- | --- | --- | --- |
|  | **antibiotic use(yes/no)** | | | **counts of antibiotics** | | | | **counts of antibiotic types** | | |  |  |
| **Antibiotic quintile** | **adjusted OR1** | **adjusted OR2** | | **adjusted OR1** | | **adjusted OR2** | | **adjusted OR1** | | **adjusted OR2** |  |  |
| no antibiotics | ref | ref | | ref | | ref | | ref | | ref |  |  |
| 1 | 1.13(1.10-1.15) | 1.00(0.98-1.02) | | 1.15(1.13-1.17) | | 1.02(1.00-1.04) | | 1.14(1.11-1.16) | | 1.01(0.99-1.03) |  |  |
| 2 | 1.23(1.20-1.26) | 1.04(1.01-1.06) | | 1.28(1.25-1.31) | | 1.07(1.04-1.10) | | 1.25(1.22-1.29) | | 1.05(1.03-1.08) |  |  |
| 3 | 1.32(1.29-1.36) | 1.08(1.05-1.11) | | 1.40(1.36-1.44) | | 1.13(1.10-1.17) | | 1.36(1.33-1.40) | | 1.10(1.07-1.13) |  |  |
| 4 | 1.43(1.40-1.46) | 1.14(1.11-1.17) | | 1.55(1.52-1.58) | | 1.22(1.19-1.25) | | 1.48(1.44-1.51) | | 1.17(1.14-1.20) |  |  |
| 5 (most frequent) | 1.60(1.56-1.64) | 1.24(1.21-1.27) | | 1.73(1.69-1.78) | | 1.33(1.30-1.37) | | 1.64(1.60-1.68) | | 1.26(1.23-1.30) |  |  |
| **Study 2: death** | | | | | | | | | | |  |  |
|  | **antibiotic use(yes/no)** | | | **counts of antibiotics** | | | | **counts of antibiotic types** | | |  |  |
| **Antibiotic quintile** | **adjusted OR1** | **adjusted OR2** | | **adjusted OR1** | | **adjusted OR2** | | **adjusted OR1** | | **adjusted OR2** |  |  |
| no antibiotics | ref | ref | | ref | | ref | | ref | | ref |  |  |
| 1 | 1.05(1.01-1.09) | 1.01(0.97-1.05) | | 1.05(1.01-1.09) | | 1.01(0.97-1.05) | | 1.05(1.01-1.09) | | 1.01(0.97-1.05) |  |  |
| 2 | 1.14(1.08-1.20) | 1.07(1.01-1.13) | | 1.14(1.08-1.20) | | 1.07(1.01-1.13) | | 1.14(1.08-1.20) | | 1.07(1.01-1.13) |  |  |
| 3 | 1.29(1.21-1.38) | 1.20(1.12-1.28) | | 1.29(1.21-1.38) | | 1.20(1.12-1.28) | | 1.29(1.21-1.38) | | 1.20(1.12-1.28) |  |  |
| 4 | 1.33(1.26-1.41) | 1.24(1.17-1.31) | | 1.33(1.26-1.41) | | 1.24(1.17-1.31) | | 1.33(1.26-1.41) | | 1.24(1.17-1.31) |  |  |
| 5 (most frequent) | 1.44(1.37-1.52) | 1.31(1.24-1.38) | | 1.42(1.35-1.50) | | 1.29(1.22-1.36) | | 1.43(1.36-1.51) | | 1.30(1.24-1.37) |  |  |
| 1. adjusted OR1: adjusted for recent 6 weeks antibiotics use | | |  | |  | |  | |  | |  |  |
| 2. adjusted OR2: adjusted for recent 6 weeks antibiotics use, as well as confounders (ethnicity, BMI category, CCI group, smoking status, IMD, care home residents, COVID-19 and flu vaccine) | | | | | | | | | | | |  |
|  |  |  |  |  |  |  |  |  |  |  |  |  |

## **Supplementary Table 5. Analysis included secondary Covid-19 outcomes & prolonged duration (90days) of same Covid-19 infection**

| **Study 1: admitted to hospitals** | | | | |
| --- | --- | --- | --- | --- |
|  | **including secondary diagnosis** | | **same Covid-19 infection (90 days)** | |
| **Antibiotic quintile l^1^** | **crude OR** | **adjusted OR^2^ (95% CI)** | **crude OR** | **adjusted OR^2^ (95% CI)** |
| no antibiotics | ref | ref | ref | ref |
| 1 | 1.22(1.20-1.24) | 1.09(1.08,1.11) | 1.24(1.21,1.26) | 1.11(1.87,1.13) |
| 2 | 1.35(1.32-1.38) | 1.15(1.27,1.18) | 1.38(1.34,1.41) | 1.17(1.14,1.20) |
| 3 | 1.49(1.146-1.52) | 1.24(1.21,1.26) | 1.62(1.58,1.66) | 1.34(1.31,1.38) |
| 4 (most frequent) | 2.01(1.97-2.05) | 1.57(1.54,1.60) | 2.20(2.14,2.25) | 1.78(1.74,1.83) |
| **Study 2: death** | | | | |
|  | **including secondary diagnosis** | | **same Covid-19 infection (90 days)** | |
| **Antibiotic quintile ^1^** | **crude OR** | **adjusted OR^2^ (95% CI)** | **crude OR** | **adjusted OR^2^ (95% CI)** |
| no antibiotics | ref | ref | ref | ref |
| 1 | 1.09(1.06-1.13) | 1.06(1.03-1.10) | 1.07(1.03,1.11) | 1.02(0.98,1.06) |
| 2 | 1.14(1.09-1.19) | 1.08(1.03-1.13) | 1.18(1.13,1.24) | 1.11(1.05.1.16) |
| 3 | 1.34(1.27-1.41) | 1.25(1.19-1.32) | 1.30(1.22,1.37) | 1.19(1.12,1.26) |
| 4 | 1.32(1.26-1.38) | 1.23(1.17-1.28) | 1.34(1.27,1.41) | 1.22(1.16,1.28) |
| 5 (most frequent) | 1.53(1.47-1.59) | 1.41(1.35-1.47) | 1.47(1.41,1.54) | 1.31(1.25,1.37) |
| 1. Antibiotic quintile means quintile groups. Study 1 only consisted of 4 antibiotic levels because the top 2 quintile groups with equal antibiotic number (=1) were merged for analysis.  2. adjusted OR: adjusted for ethnicity, BMI category, CCI group, smoking status, IMD, care home residents, COVID-19 and flu vaccine | | | | |
|  | | | | |

## **Supplementary Table 6. Analysis using different definition of severe outcome**

| **Study 1: COVID-19 death or hospital admission(case) vs. COVID-19 infection(control)** | | |
| --- | --- | --- |
| **Antibiotic quintile** | **crude OR** | **adjusted OR** |
| **no antibiotics** | **ref** | **ref** |
| **1** | 1.21 (1.18,1.23) | 1.06(1.04-1.08) |
| **2** | 1.38 (1.35,1.41) | 1.14(1.12-1.17) |
| **3** | 1.54 (1.50,1.58) | 1.22(1.19-1.25) |
| **4** | 1.78 (1.75,1.82) | 1.36(1.33-1.39) |
| **5 (most frequent)** | 2.45 (2.40,2.51) | 1.77(1.73-1.81) |

| **Study 2: COVID-19 death or ICU admission(case) vs. COVID-19 hospitalisation(control)** | | |
| --- | --- | --- |
| **Antibiotic quintile** | **crude OR** | **adjusted OR** |
| **no antibiotics** | **ref** | **ref** |
| **1** | 0.99 (0.95,1.02) | 0.96(0.93-1.00) |
| **2** | 1.06 (1.01,1.11) | 1.02(0.97-1.07) |
| **3** | 1.18 (1.12,1.25) | 1.12(1.06-1.19) |
| **4** | 1.18 (1.12,1.24) | 1.12(1.07-1.18) |
| **5 (most frequent)** | 1.25 (1.20,1.31) | 1.18(1.13-1.24) |

adjusted OR: adjusted for ethnicity, BMI category, CCI group, smoking status, IMD, care home residents, COVID-19 and flu vaccine

## **Supplementary Table 7A. Distribution of individual disease**

| **Study 1: admitted to hospitals** | | | | | |
| --- | --- | --- | --- | --- | --- |
|  | **case** | **%** | **control** | **%** | **difference** |
| Any malignancy (including leukaemia and lymphoma) | 6,975 | 7.1% | 24,300 | 4.2% | 2.9% |
| Cerebrovascular disease | 5,105 | 5.2% | 23,565 | 4.1% | 1.1% |
| Chronic pulmonary disease | 17,610 | 18.0% | 71,965 | 12.5% | 5.5% |
| Congestive heart failure | 7,760 | 7.9% | 26,005 | 4.5% | 3.4% |
| Connective tissue disease | 5,150 | 5.3% | 19,245 | 3.3% | 1.9% |
| Dementia | 7,410 | 7.6% | 43,040 | 7.5% | 0.1% |
| Diabetes | 18,950 | 19.4% | 64,245 | 11.1% | 8.2% |
| Diabetes with complications | 9,750 | 10.0% | 34,720 | 6.0% | 3.9% |
| Hemiplegia | 220 | 0.2% | 760 | 0.1% | 0.1% |
| Metastatic solid tumour | 920 | 0.9% | 2,570 | 0.4% | 0.5% |
| Mild liver disease | 595 | 0.6% | 1,475 | 0.3% | 0.4% |
| Moderate or severe liver disease | 300 | 0.3% | 835 | 0.1% | 0.2% |
| Moderate or severe renal disease | 10,855 | 11.1% | 41,445 | 7.2% | 3.9% |
| Myocardial infarction | 2,890 | 3.0% | 10,615 | 1.8% | 1.1% |
| Ulcer disease | 905 | 0.9% | 3,300 | 0.6% | 0.4% |
| Peripheral vascular disease | 2,495 | 2.5% | 9,875 | 1.7% | 0.8% |
| AIDS | 30 | 0.0% | 250 | 0.0% | 0.0% |
| **Study 2: death** | | | | | |
|  | **case** | **%** | **control** | **%** | **difference** |
| Any malignancy (including leukaemia and lymphoma) | 2,340 | 10.5% | 11,085 | 8.7% | 1.8% |
| Cerebrovascular disease | 1,795 | 8.0% | 9,640 | 7.6% | 0.5% |
| Chronic pulmonary disease | 4,120 | 18.5% | 22,265 | 17.5% | 1.0% |
| Congestive heart failure | 3,200 | 14.3% | 13,845 | 10.9% | 3.4% |
| Connective tissue disease | 1,560 | 7.0% | 7,570 | 6.0% | 1.0% |
| Dementia | 3,210 | 14.4% | 15,225 | 12.0% | 2.4% |
| Diabetes | 5,235 | 23.4% | 26,675 | 21.0% | 2.5% |
| Diabetes with complications | 2,970 | 13.3% | 14,950 | 11.8% | 1.6% |
| Hemiplegia | 75 | 0.3% | 375 | 0.3% | 0.0% |
| Metastatic solid tumour | 365 | 1.6% | 1,235 | 1.0% | 0.7% |
| Mild liver disease | 210 | 0.9% | 580 | 0.5% | 0.5% |
| Moderate or severe liver disease | 115 | 0.5% | 245 | 0.2% | 0.3% |
| Moderate or severe renal disease | 4,010 | 18.0% | 18,820 | 14.8% | 3.2% |
| Myocardial infarction | 1,070 | 4.8% | 4,975 | 3.9% | 0.9% |
| Ulcer disease | 330 | 1.5% | 1,450 | 1.1% | 0.3% |
| Peripheral vascular disease | 965 | 4.3% | 4,460 | 3.5% | 0.8% |
| AIDS | <5 | 0.0% | 10 | 0.0% | 0.0% |

The counts of patients were round to nearest 5 number in line with disclosure controls.

## **Supplementary Table 7B. Analysis adjusted for individual disease**

| **Study 1: admitted to hospitals** | | |  |
| --- | --- | --- | --- |
| **Antibiotic quintile** | **adjusted OR1 (95% CI)** | **adjusted OR2 (95% CI)** |  |
| no antibiotics | ref | ref |  |
| 1 | 1.11(1.09-1.13) | 1.05(1.03-1.07) |  |
| 2 | 1.22(1.19-1.25) | 1.13(1.10-1.16) |  |
| 3 | 1.33(1.29-1.37) | 1.23(1.19-1.26) |  |
| 4 | 1.47(1.44-1.51) | 1.37(1.34-1.40) |  |
| 5 (most frequent) | 1.95(1.91-2.00) | 1.84(1.80-1.89) |  |
| **Study 2: death** | | |  |
| **Antibiotic quintile** | **adjusted OR1 (95% CI)** | **adjusted OR2 (95% CI)** |  |
| no antibiotics | ref | ref |  |
| 1 | 1.02(0.98-1.06) | 1.12(1.06-1.19) |  |
| 2 | 1.10(1.05-1.16) | 1.16(1.09-1.24) |  |
| 3 | 1.25(1.17-1.33) | 1.29(1.20-1.38) |  |
| 4 | 1.30(1.23-1.37) | 1.32(1.24-1.40) |  |
| 5 (most frequent) | 1.40(1.33-1.47) | 1.38(1.31-1.45) |  |
| 1. adjusted OR1: adjusted for individual disease 2. adjusted OR2: adjusted for individual disease, as well as confounders (ethnicity, BMI category, smoking status, IMD, care home residents, COVID-19 and flu vaccine) | | |  |
|  | | |  |

## **Supplementary Table 8. Comparison of matched and non-matched cases**

| **Study 1: admitted to hospitals** | | |
| --- | --- | --- |
|  | **Non-matched** | **matched** |
| **Number of patients** | 545 | 97,880 |
| **mean age (SD)** | 85.60 (12.59) | 65.60 (17.64) |
| **sex (male%)** | 330 (60%) | 54,270 (55%) |
| **Study 2: death** | | |
|  | **Non-matched** | **matched** |
| **Number of patients** | 330 | 22,330 |
| **mean age (SD)** | 86.98 (13.87) | 77.10 (12.14) |
| **sex (male%)** | 180 (55%) | 13,500 (60%) |

The counts of patients were round to nearest 5 number in line with disclosure controls.

## **Supplementary Table 9. Complete case analysis**

| **Study 1: admitted to hospitals** | | |
| --- | --- | --- |
| **Antibiotic quintile** | **crude OR** | **adjusted OR1** |
| **no antibiotics** | **ref** | **ref** |
| **1** | 1.07(1.04-1.09) | 1.02(1.00-1.05) |
| **2** | 1.17(1.13-1.20) | 1.09(1.05-1.12) |
| **3** | 1.28(1.23-1.32) | 1.16(1.12-1.20) |
| **4** | 1.42(1.39-1.46) | 1.28(1.25-1.32) |
| **5 (most frequent)** | 2.01(1.95-2.06) | 1.74(1.69-1.79) |
| **Study 2: death** | | |
| **Antibiotic quintile** | **crude OR** | **adjusted OR1** |
| **no antibiotics** | **ref** | **ref** |
| **1** | 1.06 (0.99,1.12) | 1.03(0.99-1.08) |
| **2** | 1.13 (1.04,1.22) | 1.14(1.07-1.21) |
| **3** | 1.23 (1.11,1.36) | 1.33(1.23-1.43) |
| **4** | 1.32 (1.21,1.44) | 1.34(1.26-1.43) |
| **5 (most frequent)** | 1.49 (1.39,1.61) | 1.49(1.41-1.57) |

1.adjusted OR: adjusted for ethnicity, BMI category, CCI group, smoking status, IMD, care home residents, COVID-19 and flu vaccine

## **Supplementary Table 10. Code lists used for variable definition**

1. Code lists are available at <https://www.opencodelists.org/>
2. Link: <https://www.opencodelists.org/codelist/> add **code list version**

| Variable type | variable | **Code list version** |
| --- | --- | --- |
| Outcome | COVID-19 codes in primary care | opensafely/covid-identification-in-primary-care-probable-covid-clinical-code/24391856  opensafely/covid-identification-in-primary-care-probable-covid-positive-test/3d488b8b  opensafely/covid-identification-in-primary-care-probable-covid-sequelae/0b29a521 |
| Exposure | All antibiotics | user/BillyZhongUOM/brit_new_dmd/792101bd |
| Exposure | 79 types of antibiotics | user/yayang/codes_ab_type_amikacincsv/1541da32  user/yayang/codes_ab_type_amoxicillincsv/7c3266fc  user/yayang/codes_ab_type_ampicillincsv/6322f3c7  user/yayang/codes_ab_type_azithromycincsv/4a138092  user/yayang/codes_ab_type_aztreonamcsv/31040d5d  user/yayang/codes_ab_type_benzylpenicillincsv/17f49a28  user/yayang/codes_ab_type_cefaclorcsv/7ee526f2  user/yayang/codes_ab_type_cefadroxilcsv/65d5b3bd  user/yayang/codes_ab_type_cefalexincsv/4cc64088  user/yayang/codes_ab_type_cefamandolecsv/33b6cd53  user/yayang/codes_ab_type_cefazolincsv/1aa75a1e  user/yayang/codes_ab_type_cefepimecsv/0197e6e9  user/yayang/codes_ab_type_cefiximecsv/688873b3  user/yayang/codes_ab_type_cefotaximecsv/4f79007e  user/yayang/codes_ab_type_cefoxitincsv/36698d49  user/yayang/codes_ab_type_cefpiromecsv/1d5a1a14  user/yayang/codes_ab_type_cefpodoximecsv/6b3b33a9  user/yayang/codes_ab_type_cefprozilcsv/522bc074  user/yayang/codes_ab_type_cefradinecsv/391c4d3f  user/yayang/codes_ab_type_ceftazidimecsv/200cda0a  user/yayang/codes_ab_type_ceftriaxonecsv/06fd66d5  user/yayang/codes_ab_type_cefuroximecsv/6dedf39f  user/yayang/codes_ab_type_chloramphenicolcsv/54de806a  user/yayang/codes_ab_type_cilastatincsv/3bcf0d35  user/yayang/codes_ab_type_ciprofloxacincsv/22bf9a00  user/yayang/codes_ab_type_clarithromycincsv/09b026cb  user/yayang/codes_ab_type_clindamycincsv/70a0b395  user/yayang/codes_ab_type_co-amoxiclavcsv/57914060  user/yayang/codes_ab_type_co-fluampicilcsv/3e81cd2b  user/yayang/codes_ab_type_colistimethatecsv/257259f6  user/yayang/codes_ab_type_dalbavancincsv/0c62e6c1  user/yayang/codes_ab_type_dalfopristincsv/5a440056  user/yayang/codes_ab_type_daptomycincsv/41348d21  user/yayang/codes_ab_type_demeclocyclinecsv/282519ec  user/yayang/codes_ab_type_doripenemcsv/0f15a6b7  user/yayang/codes_ab_type_doxycyclinecsv/76063381  user/yayang/codes_ab_type_ertapenemcsv/5cf6c04c  user/yayang/codes_ab_type_erythromycincsv/43e74d17  user/yayang/codes_ab_type_fidaxomicincsv/2ad7d9e2  user/yayang/codes_ab_type_flucloxacillincsv/11c866ad  user/yayang/codes_ab_type_fosfomycincsv/78b8f377  user/yayang/codes_ab_type_fusidatecsv/5fa98042  user/yayang/codes_ab_type_gentamicincsv/469a0d0d  user/yayang/codes_ab_type_levofloxacincsv/2d8a99d8  user/yayang/codes_ab_type_linezolidcsv/147b26a3  user/yayang/codes_ab_type_lymecyclinecsv/7b6bb36d  user/yayang/codes_ab_type_meropenemcsv/625c4038  user/yayang/codes_ab_type_methenaminecsv/494ccd03  user/yayang/codes_ab_type_metronidazolecsv/303d59ce  user/yayang/codes_ab_type_minocyclinecsv/7e1e7363  user/yayang/codes_ab_type_moxifloxacincsv/650f002e  user/yayang/codes_ab_type_nalidixic-acidcsv/4bff8cf9  user/yayang/codes_ab_type_neomycincsv/32f019c4  user/yayang/codes_ab_type_netilmicincsv/19e0a68f  user/yayang/codes_ab_type_nitazoxanidcsv/00d1335a  user/yayang/codes_ab_type_nitrofurantoincsv/67c1c024  user/yayang/codes_ab_type_norfloxacincsv/4eb24cef  user/yayang/codes_ab_type_ofloxacincsv/35a2d9ba  user/yayang/codes_ab_type_oxytetracyclinecsv/1c936685  user/yayang/codes_ab_type_phenoxymethylpenicillincsv/0383f350  user/yayang/codes_ab_type_piperacillincsv/6a74801a  user/yayang/codes_ab_type_pivmecillinamcsv/51650ce5  user/yayang/codes_ab_type_pristinamycincsv/385599b0  user/yayang/codes_ab_type_rifaximincsv/1f46267b  user/yayang/codes_ab_type_sulfadiazinecsv/0636b346  user/yayang/codes_ab_type_sulfamethoxazolecsv/6d274010  user/yayang/codes_ab_type_sulfapyridinecsv/5417ccdb  user/yayang/codes_ab_type_taurolidincsv/3b0859a6  user/yayang/codes_ab_type_tedizolidcsv/21f8e671  user/yayang/codes_ab_type_teicoplanincsv/08e9733c  user/yayang/codes_ab_type_telithromycincsv/6fda0006  user/yayang/codes_ab_type_temocillincsv/2cb088e8  user/yayang/codes_ab_type_tetracyclinecsv/56ca8cd1  user/yayang/codes_ab_type_ticarcillincsv/3dbb199c  user/yayang/codes_ab_type_tigecyclinecsv/24aba667  user/yayang/codes_ab_type_tinidazolecsv/0b9c3332  user/yayang/codes_ab_type_tobramycincsv/728cbffc  user/yayang/codes_ab_type_trimethoprimcsv/597d4cc7  user/yayang/codes_ab_type_vancomycincsv/406dd992 |
| Confounder | Charlson Comobidities | user/yayang/charlson01_cancer/796c49a5  user/yayang/charlson02_cvd/605cd670  user/yayang/charlson03_copd/474d633b  user/yayang/charlson04_heart_failure/2e3df006  user/yayang/charlson05_connective_tissue/152e7cd1  user/yayang/charlson06_dementia/7c1f099b  user/yayang/charlson07_diabetes/630f9666  user/yayang/charlson08_diabetes_with_complications/4a002331  user/yayang/charlson09_hemiplegia/30f0affc  user/yayang/charlson10_hiv/17e13cc7  user/yayang/charlson11_metastatic_cancer/7ed1c991  user/yayang/charlson12_mild_liver/65c2565c  user/yayang/charlson13_mod_severe_liver/4cb2e327  user/yayang/charlson14_moderate_several_renaldiseae/33a36ff2  user/yayang/charlson15_mi/1a93fcbd  user/yayang/charlson16_peptic_ulcer/36562fe8  user/yayang/charlson17_peripheral_vascular/68751652 |
| Confounder | Care home residency | primis-covid19-vacc-uptake/longres/v1 |
| Confounder | BMI | primis-covid19-vacc-uptake/bmi_stage/261252c3 |
| Confounder | Smoking | opensafely/smoking-clear/10307fc4  opensafely/smoking-unclear/77210c8e |
| Confounder | Flu vaccination | opensafely/influenza-vaccination/01ec0c67  opensafely/influenza-vaccination-clinical-codes-given/443b7295 |

#

# **Supplementary Figures**

## **Supplementary Figure 1. Patient selection criteria and variable measurement**

1. Cohort selection, confounding and exposure variables measurements
2. Case and control selection criteria for study 1; Case index date was identified from SUS; Control index date was identified from GP and SGSS
3. Case and control selection criteria for study 2; Index date was identified from SUS for both case and control groups.

a. Patients inclusion criteria


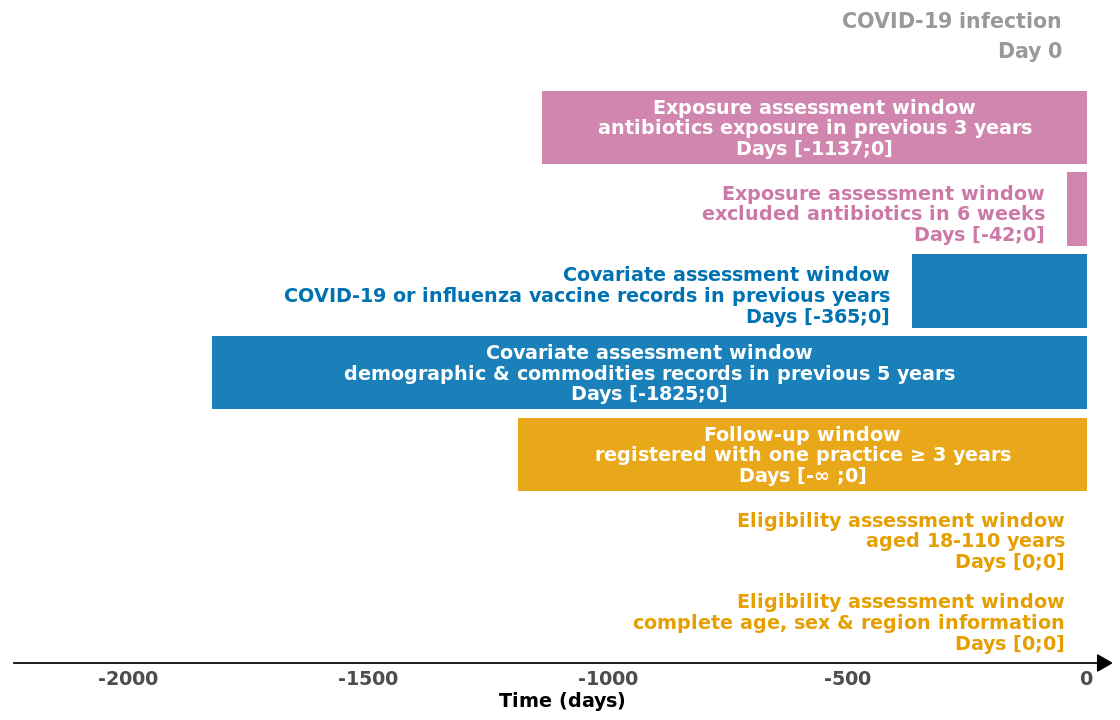


- The code lists used for variables definition could be found in Supplementary Table 9.

b. Case and Control criteria: Study 1

- COVID-19 infection Day 0 (index date)
  - case: incident COVID-19 hospital admissions
    - ICD-10 U07.1 and U07.2 as primary diagnosis identified form SUS
  - control: incident COVID-19 positive patients
    - positive COVID-19 test result identified from SGSS
    - COVID-19 related codes identified from GP, published by OpenSAFELY team, included (code lists as Supplementary Table 9):
      - probable COVID-19 clinical code
      - probable COVID-19 positive test
      - probable COVID-19 sequelae
- Exclusion for COVID-19 history
  - Case: COVID-19 codes found until 30 days before index date (SGSS or GP records found within 30 days prior to index date was regarded same infection episode)
  - Control: COVID-19 codes found before index date
  - exclusion criteria as below:
    - positive COVID-19 test results identified from SGSS
    - COVID-19 related codes identified from GP
    - COVID-19 hospital admissions from SUS, not restricted to primary diagnosis
    - COVID-19 death certifications from ONS, not restricted to underlying cause
    - COVID-19 in-hospital deaths found from CPNS
- Exclusion for severe COVID-19 case for controls
  - COVID-19 hospital admissions from SUS, not restricted to primary diagnosis
  - COVID-19 death certifications from ONS, not restricted to underlying cause
  - COVID-19 in-hospital deaths found from CPNS
- The code lists used for variables definition could be found in Supplementary Table 9.

c. Case and Control criteria: Study 2

- COVID-19 infection Day 0 (index date of incident COVID-19 hospital admission)
  - case: with severe COVID-19 outcome
  - control: without severe COVID-19 outcome
  - severe outcomes were defined as below
    - COVID-19 death certifications from ONS, not restricted to underlying cause
    - COVID-19 in-hospital deaths found from CPNS
- Exclusion for COVID-19 history in both cases and controls
  - exclusion criteria as below:
    - positive COVID-19 test results identified from SGSS
    - COVID-19 related codes identified from GP
    - COVID-19 hospital admissions from SUS, not restricted to primary diagnosis
    - COVID-19 death certifications from ONS, not restricted to underlying cause
    - COVID-19 in-hospital deaths found from CPNS

## Supplementary Figure 2. Analysis adjusted for timing of antibiotics prescribed

| **A. Study 1: admitted to hospitals** |
| --- |
| 1. excluding antibiotics in 6 weeks |
| 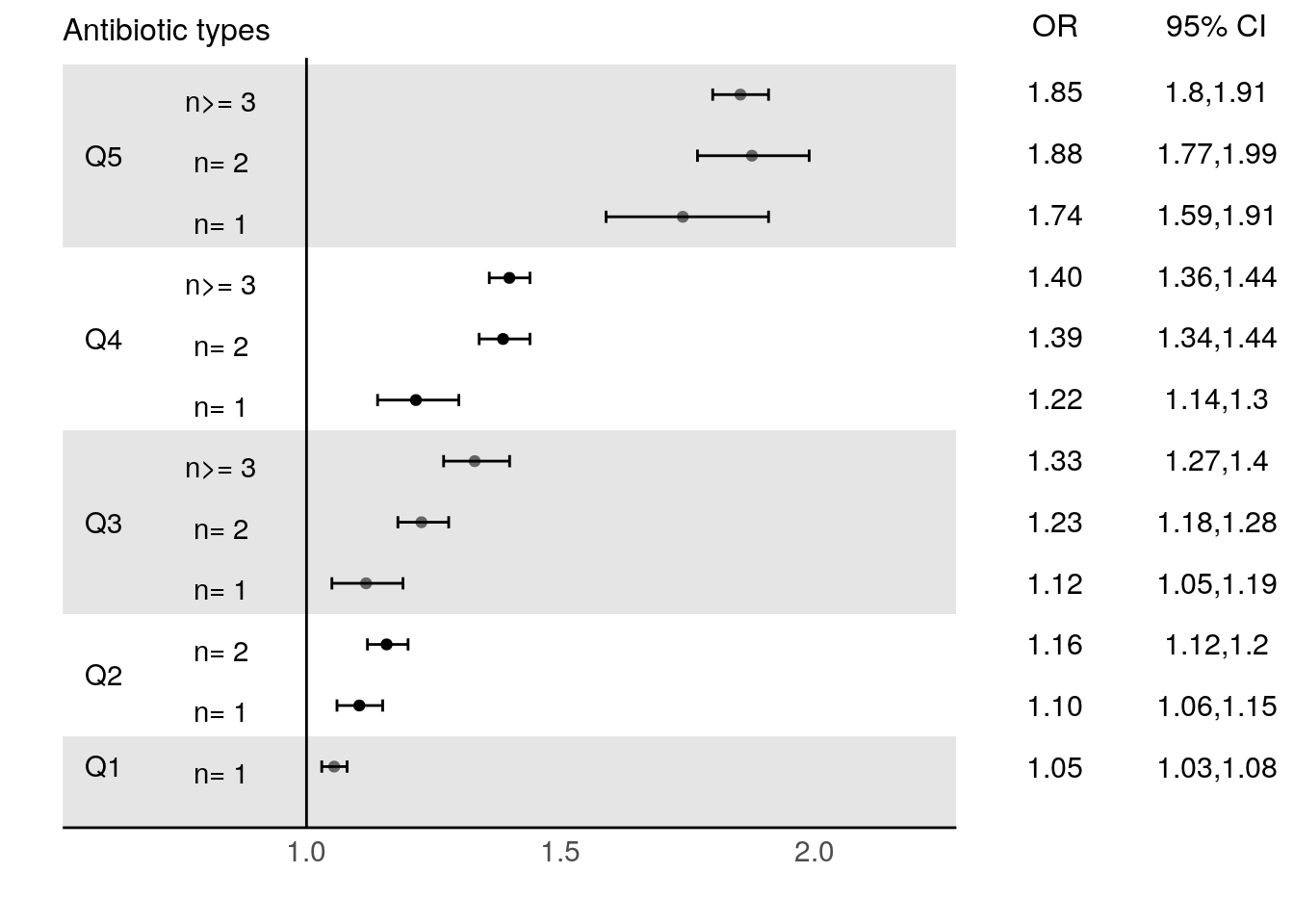 |
| 2. including antibiotics in 6 weeks |
| 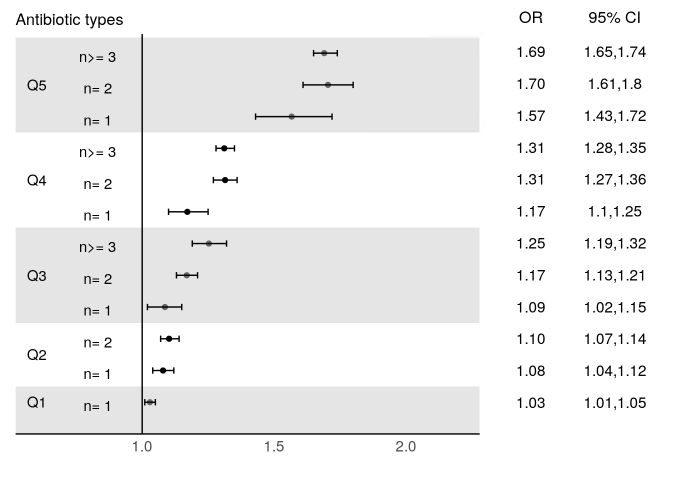 |

*OR: adjusted for timing of latest antibiotics, ethnicity, BMI category, CCI group, smoking status, IMD, care home residents, COVID-19 and flu vaccine

* Adjusted ORs for COVID-19 outcomes stratified by number of antibiotic types in the 3 years by quintile (Q1-Q5) of total number of prior antibiotic prescription

| **B. Study 2: death** |
| --- |
| 1. excluding antibiotics in 6 weeks |
| 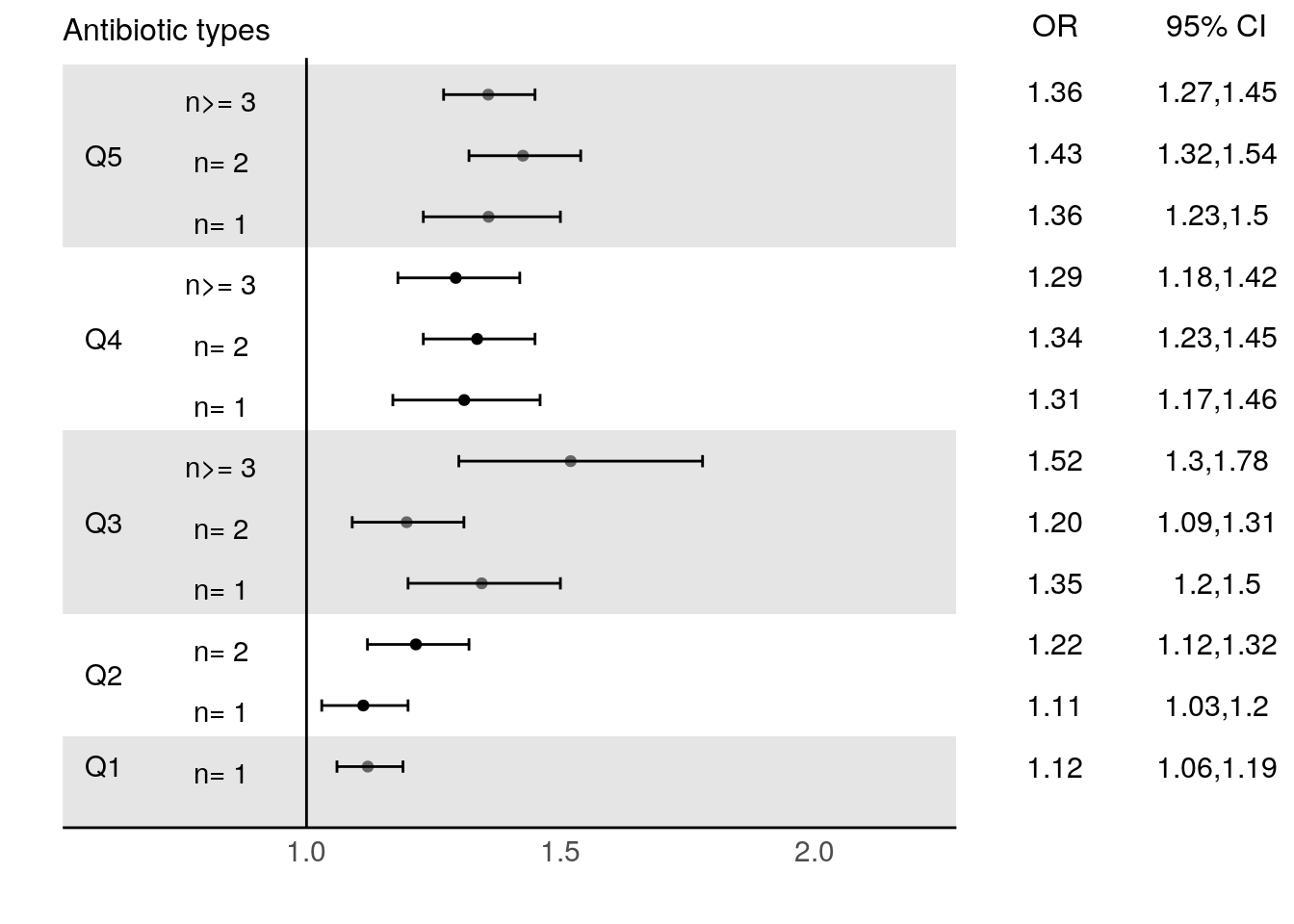 |
| 2. including antibiotics in 6 weeks |
| 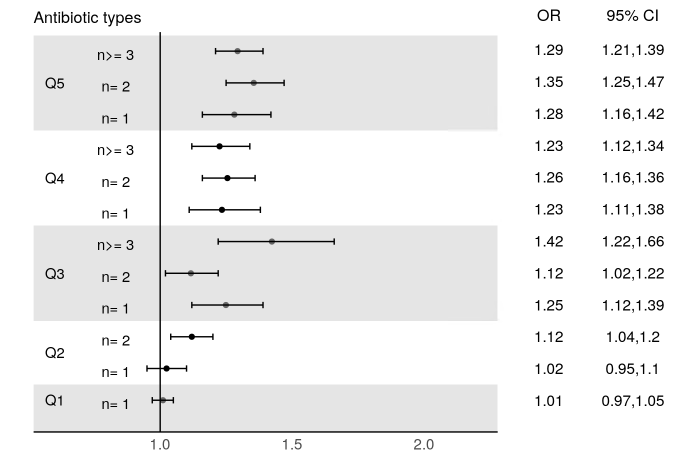 |

*OR: adjusted for timing of latest antibiotics, ethnicity, BMI category, CCI group, smoking status, IMD, care home residents, COVID-19 and flu vaccine

* Adjusted ORs for COVID-19 outcomes stratified by number of antibiotic types in the 3 years by quintile (Q1-Q5) of total number of prior antibiotic prescription

## Supplementary Figure 3. Analysis removing outliers

| **A. Study 1: admitted to hospitals** |
| --- |
| 1. excluding patients whose antibiotics greater than 99.99^th^ percentile (value=221) |
| 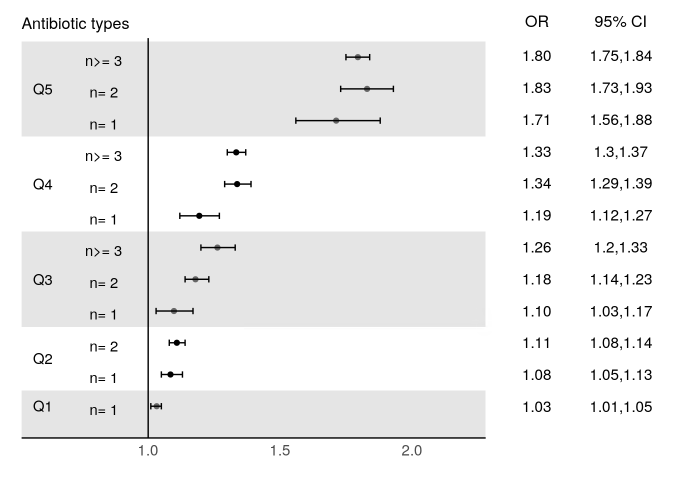 |
| 2. excluding patients whose antibiotics greater than 99^th^ percentile (value=45) |
| 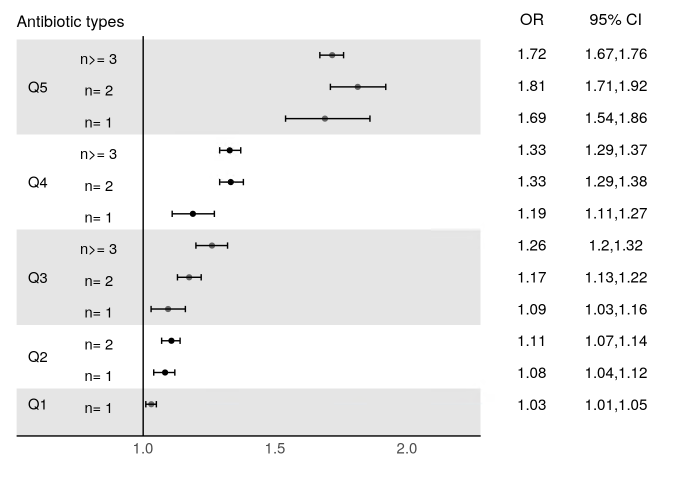 |
| 3. excluding patients whose antibiotics greater than 90^th^ percentile (value=11) |
| 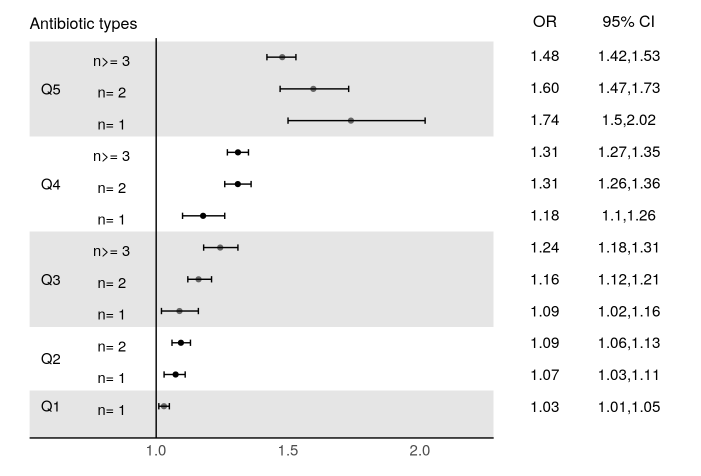 |

*OR were adjusted for ethnicity, BMI category, CCI group, smoking status, IMD, care home residents, COVID-19 and flu vaccine

* Adjusted ORs for COVID-19 outcomes stratified by number of antibiotic types in the 3 years by quintile (Q1-Q5) of total number of prior antibiotic prescription

| **B. Study 2: death** |
| --- |
| 1. excluding patients whose antibiotics greater than 99.99^th^ percentile (value=244) |
| 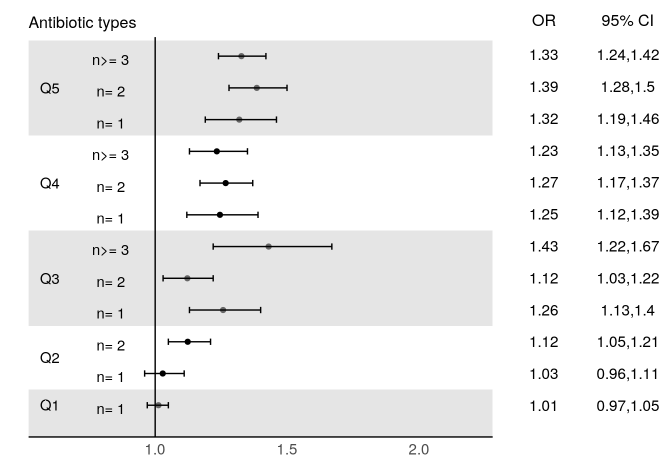 |
| 2. excluding patients whose antibiotics greater than 99^th^ percentile (value=53) |
| 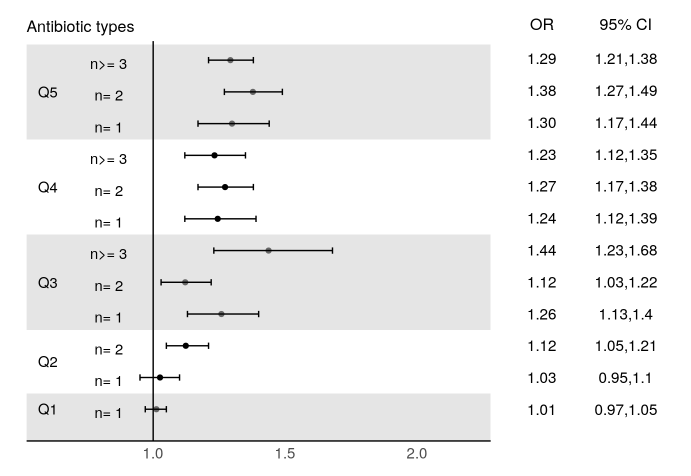 |
| 3. excluding patients whose antibiotics greater than 90^th^ percentile (value=13) |
| 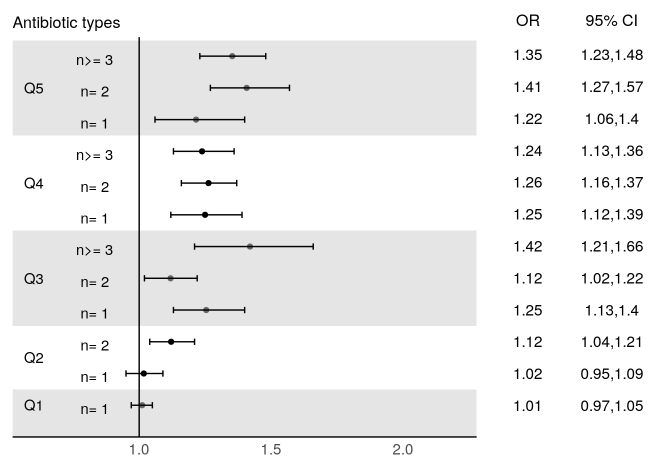 |

*OR were adjusted for ethnicity, BMI category, CCI group, smoking status, IMD, care home residents, COVID-19 and flu vaccine

* Adjusted ORs for COVID-19 outcomes stratified by number of antibiotic types in the 3 years by quintile (Q1-Q5) of total number of prior antibiotic prescription

## Supplementary Figure 4. Analysis adjusted for antibiotics in recent 6 weeks

| **A. Study 1: admitted to hospitals** |
| --- |
| 1. antibiotic use(yes/no) |
| 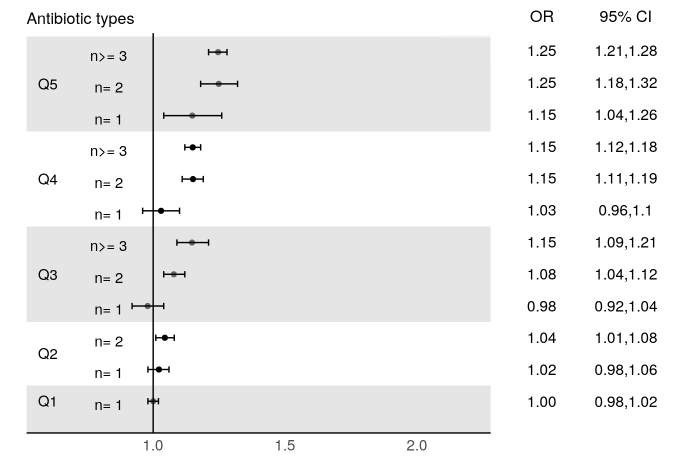 |
| 2. counts of antibiotics |
| 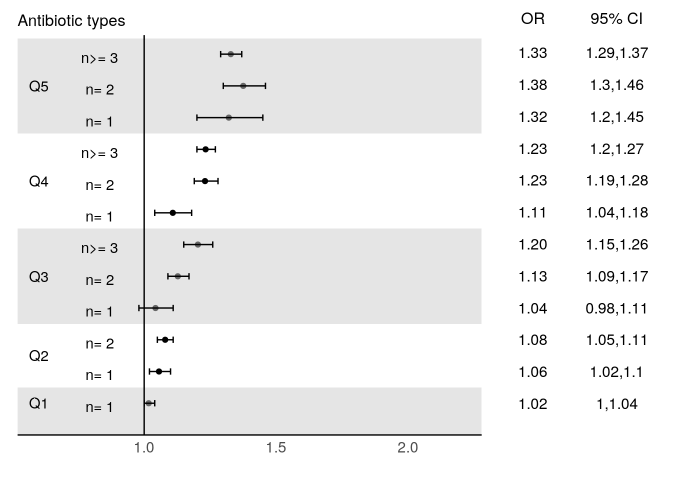 |
| 3. counts of antibiotic types |
| 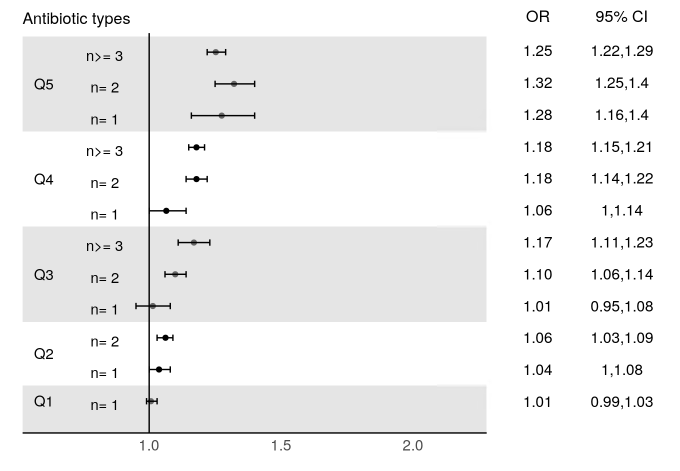 |

*OR were adjusted for ethnicity, BMI category, CCI group, smoking status, IMD, care home residents, COVID-19 and flu vaccine

* Adjusted ORs for COVID-19 outcomes stratified by number of antibiotic types in the 3 years by quintile (Q1-Q5) of total number of prior antibiotic prescription

| **B. Study 2: death** |
| --- |
| 1. antibiotic use(yes/no) |
| 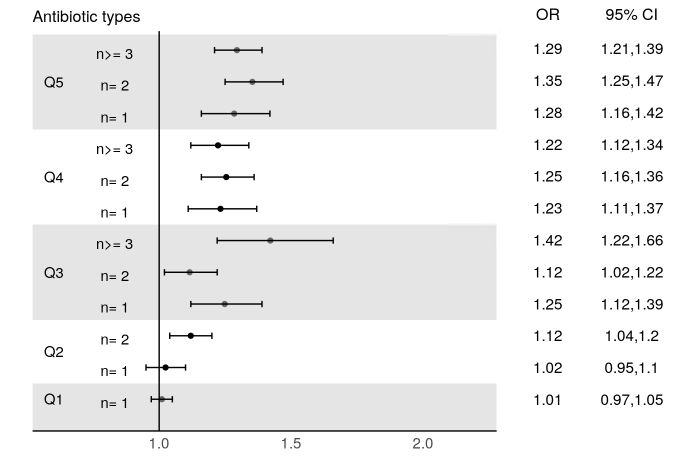 |
| 2. counts of antibiotics |
| 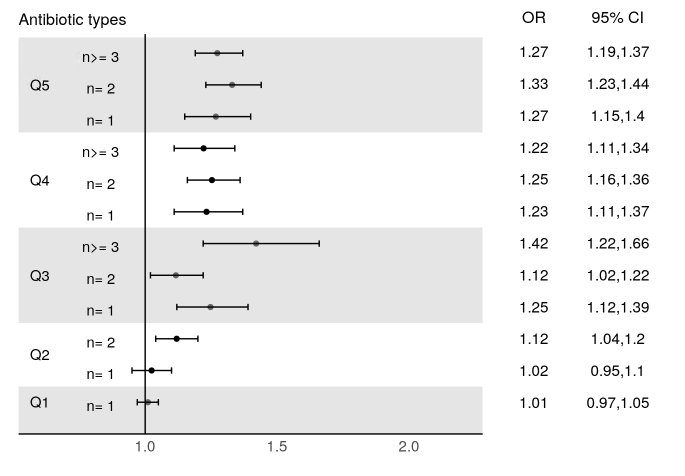 |
| 3. counts of antibiotic types |
| 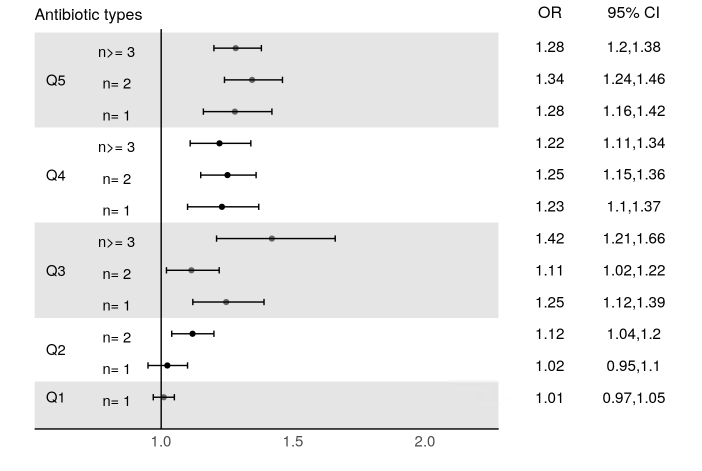 |

*OR were adjusted for ethnicity, BMI category, CCI group, smoking status, IMD, care home residents, COVID-19 and flu vaccine

* Adjusted ORs for COVID-19 outcomes stratified by number of antibiotic types in the 3 years by quintile (Q1-Q5) of total number of prior antibiotic prescription

## Supplementary Figure 5. Analysis included secondary Covid-19 outcomes & prolonged duration (90days) of same Covid-19 infection

| **A. Study 1: admitted to hospitals** |
| --- |
| 1. including secondary diagnosis |
| 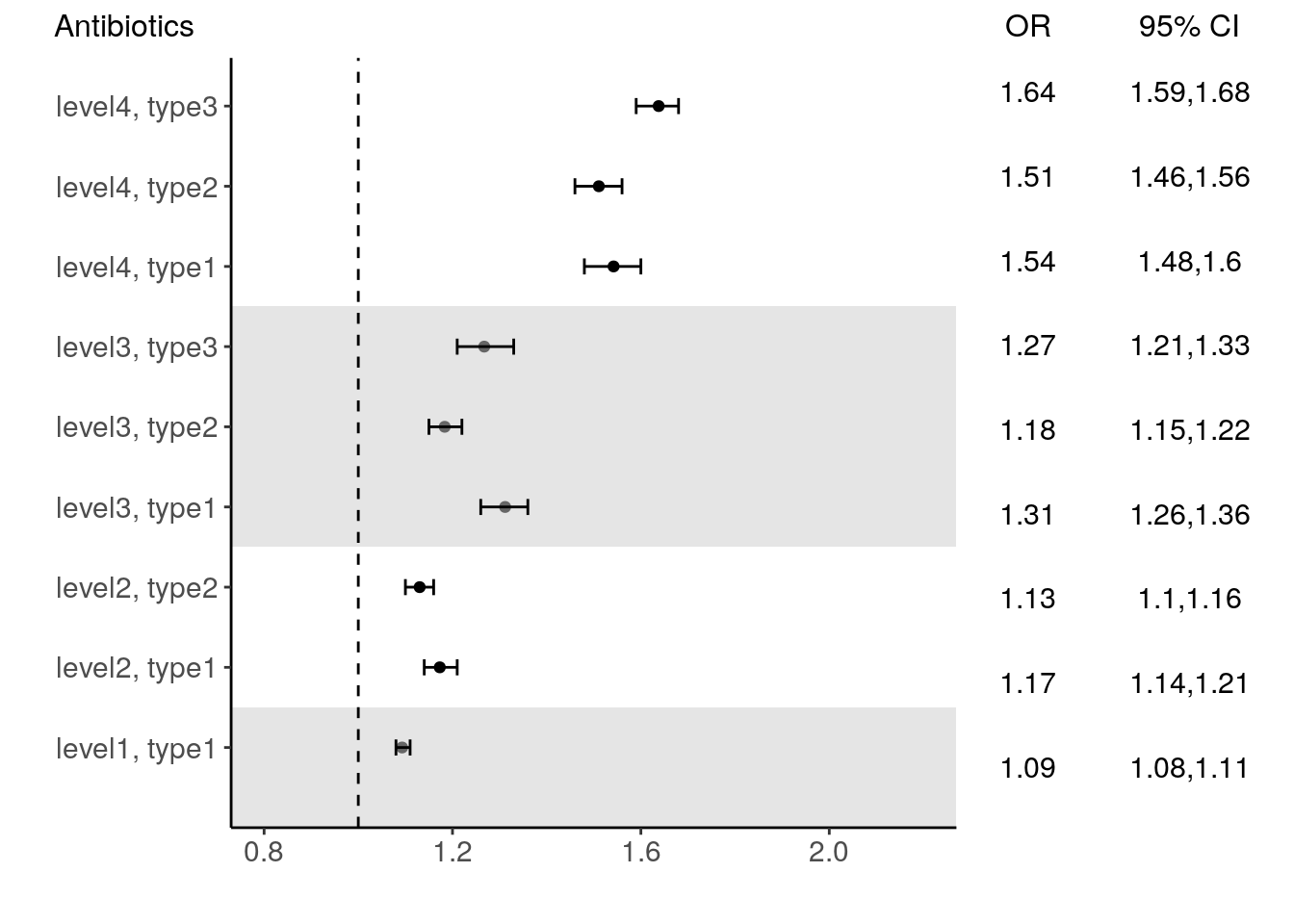 |
| 2. same infection (90 days) |
| 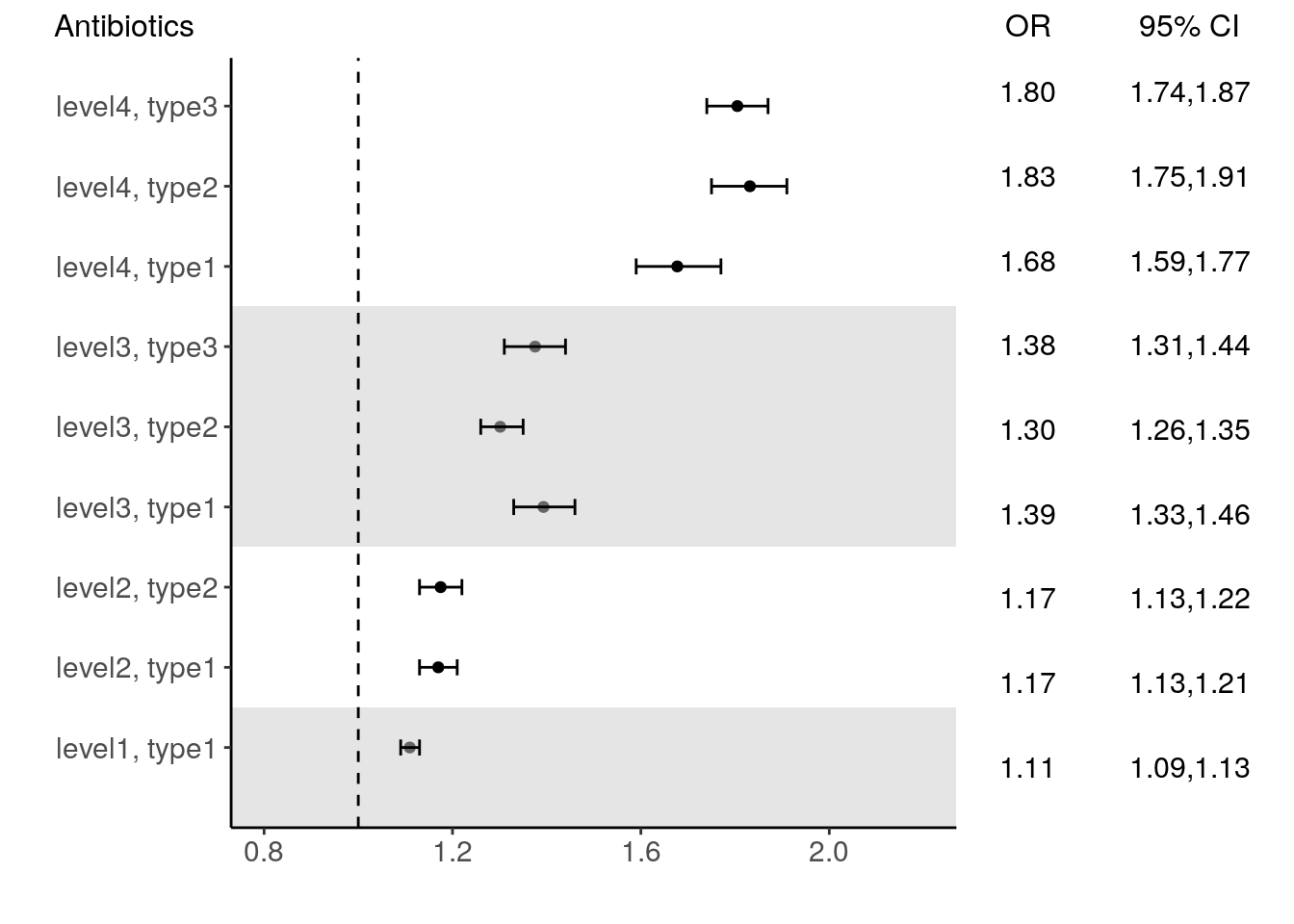 |

1. Antibiotics level means quintile groups which was stratified by number of antibiotic types in the 3 years. Study 1 only consisted of 4 antibiotics levels because the top 2 quintile groups with equal antibiotic number (=1) were merged for analysis.
2. type1 means 1 antibiotic type, type2 means 2 antibiotic types, type3 means more than 3 antibiotic types
3. OR were adjusted for ethnicity, BMI category, CCI group, smoking status, IMD, care home residents, COVID-19 and flu vaccine

| **B. Study 2: death** |
| --- |
| 1. including secondary diagnosis |
| 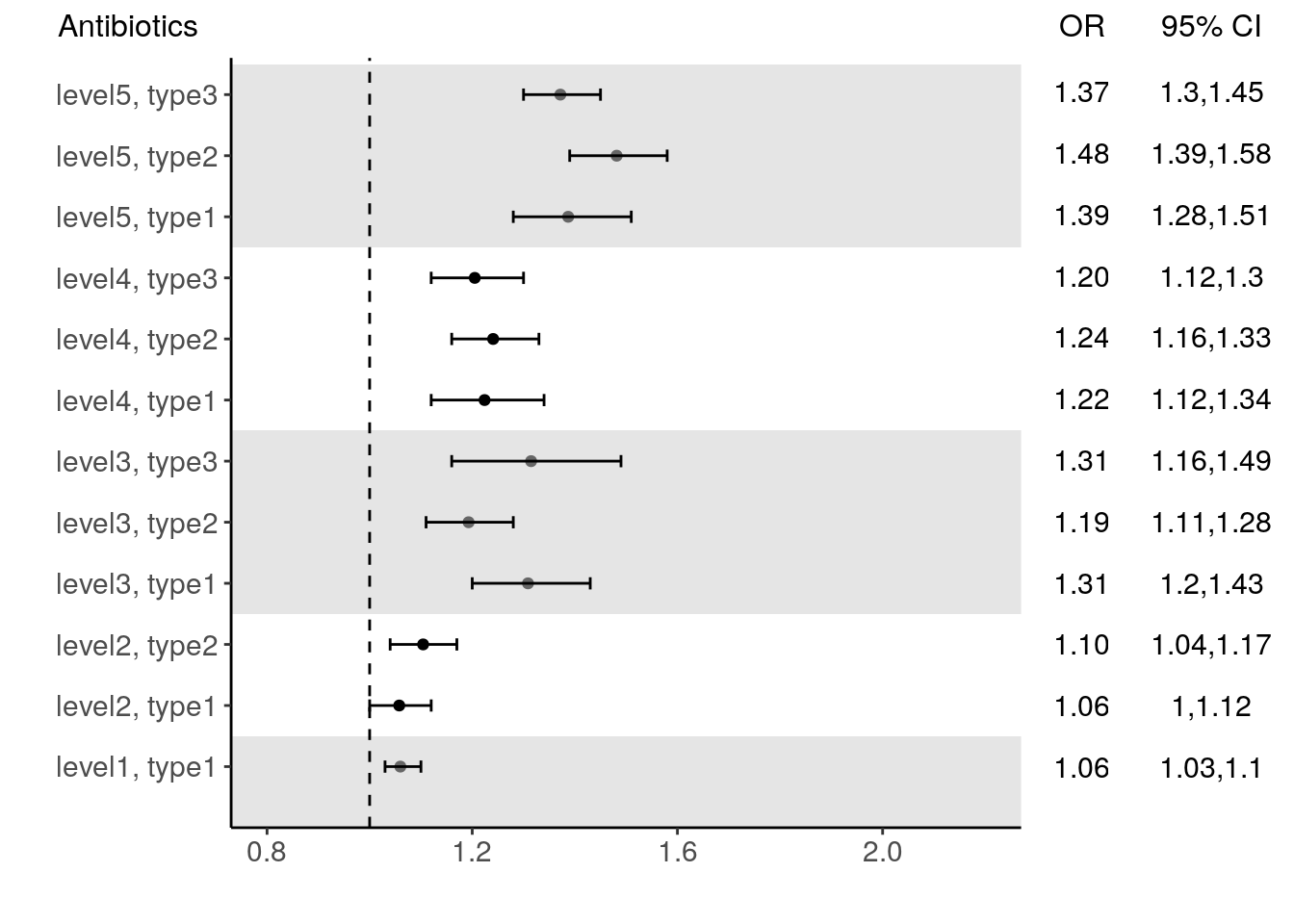 |
| 2. same infection (90 days) |
| 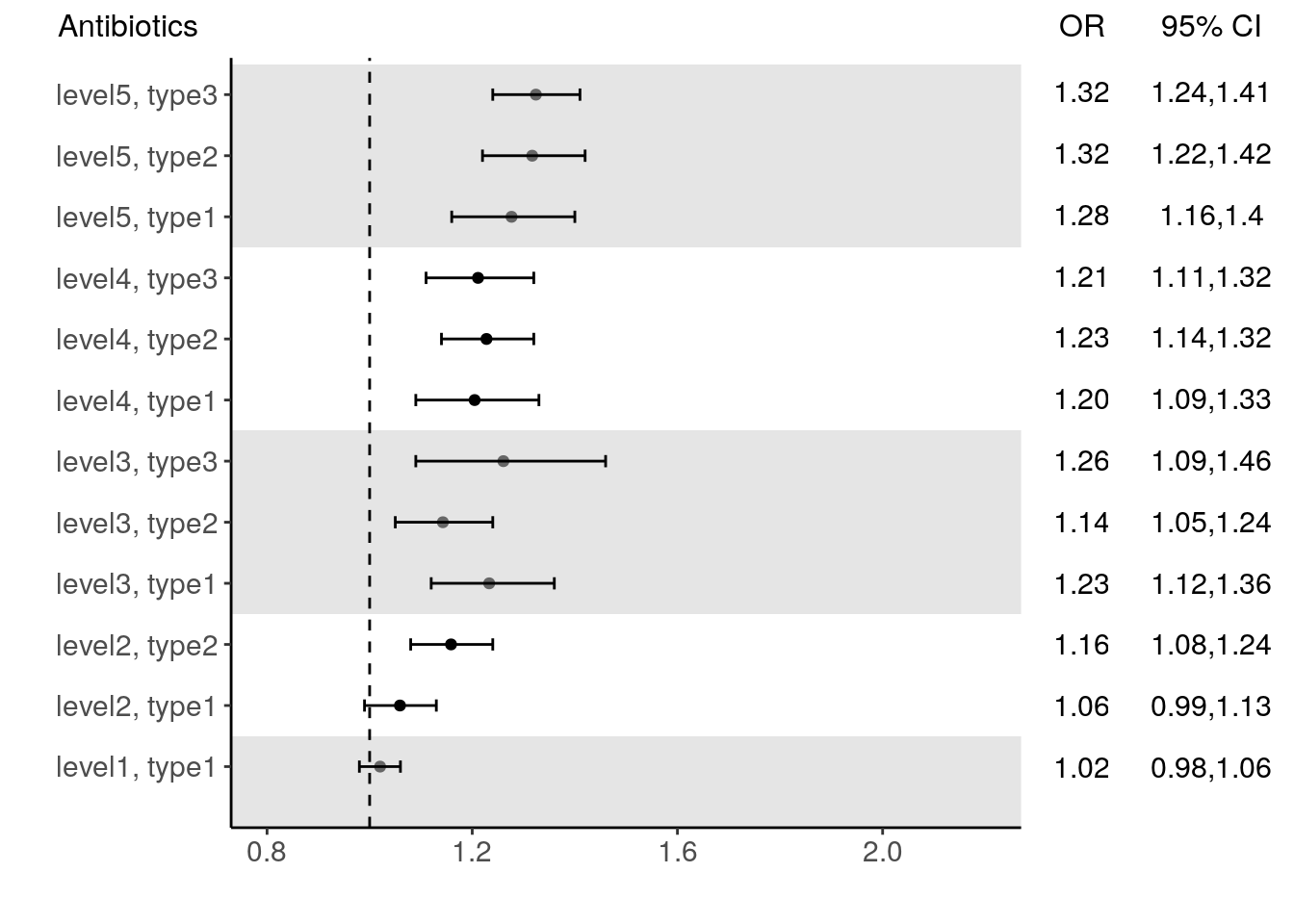 |

1. Antibiotics level means quintile groups which was stratified by number of antibiotic types in the 3 years.
2. type1 means 1 antibiotic type, type2 means 2 antibiotic types, type3 means more than 3 antibiotic types
3. OR were adjusted for ethnicity, BMI category, CCI group, smoking status, IMD, care home residents, COVID-19 and flu vaccine

## Supplementary Figure 6. Analysis using different definition of severe outcome

| **A. Study 1: admitted to hospitals or death** |
| --- |
| 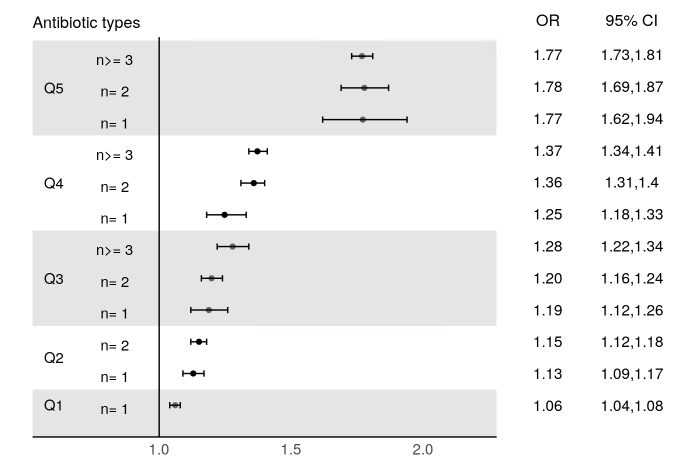 |
| **B. Study 2: admitted to ICU or death** |
| 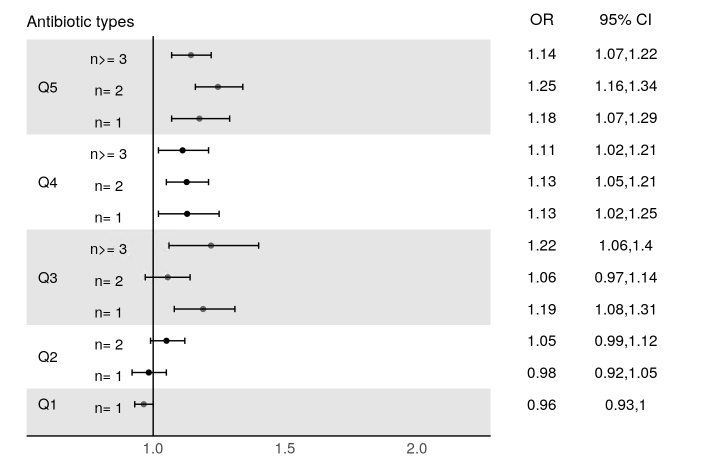 |

*OR were adjusted for ethnicity, BMI category, CCI group, smoking status, IMD, care home residents, COVID-19 and flu vaccine

* Adjusted ORs for COVID-19 outcomes stratified by number of antibiotic types in the 3 years by quintile (Q1-Q5) of total number of prior antibiotic prescription

## Supplementary Figure 7. Analysis adjusted for individual disease

| **A. Study 1: admitted to hospitals** |
| --- |
| 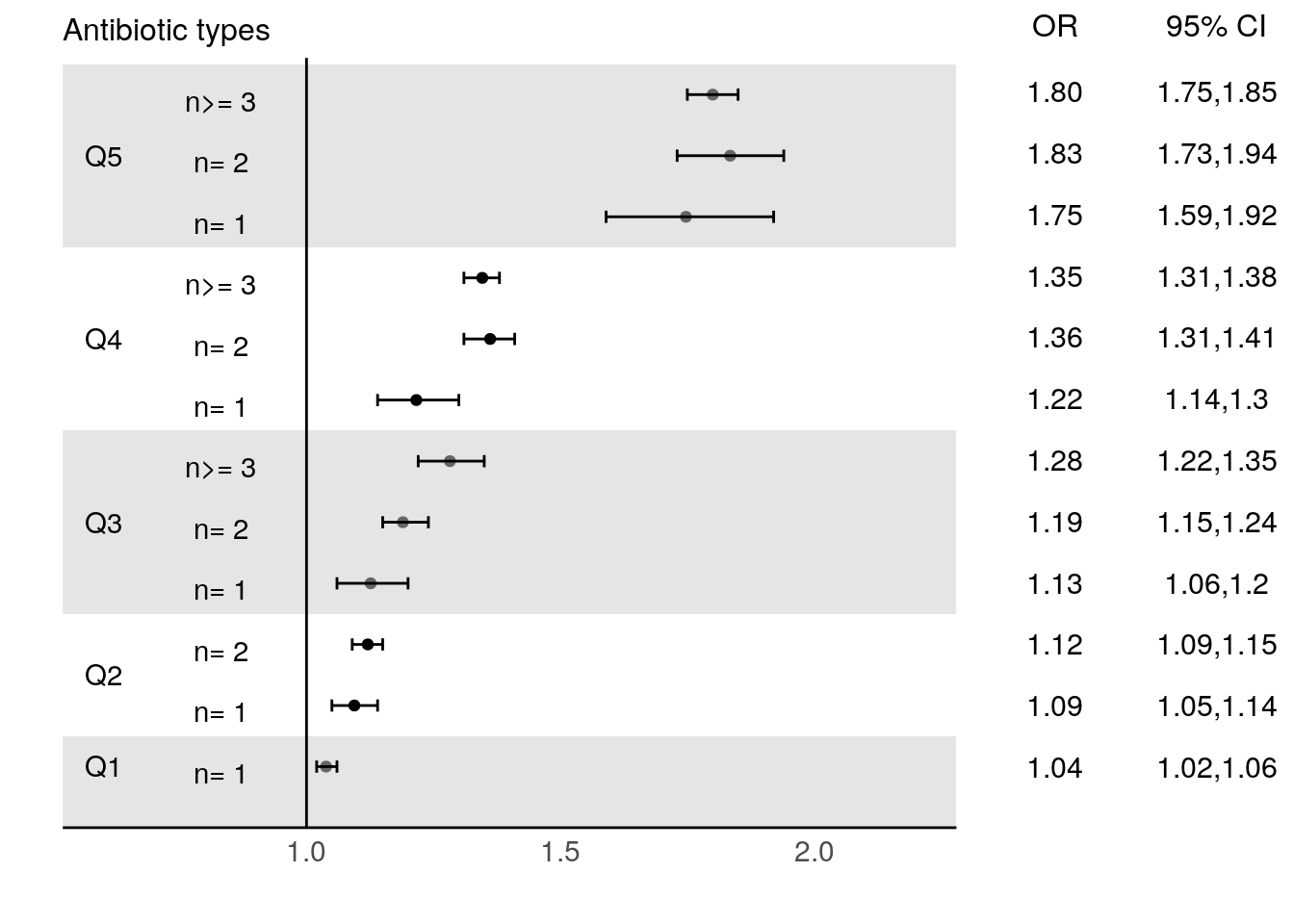 |
| **B. Study 2: death** |
| 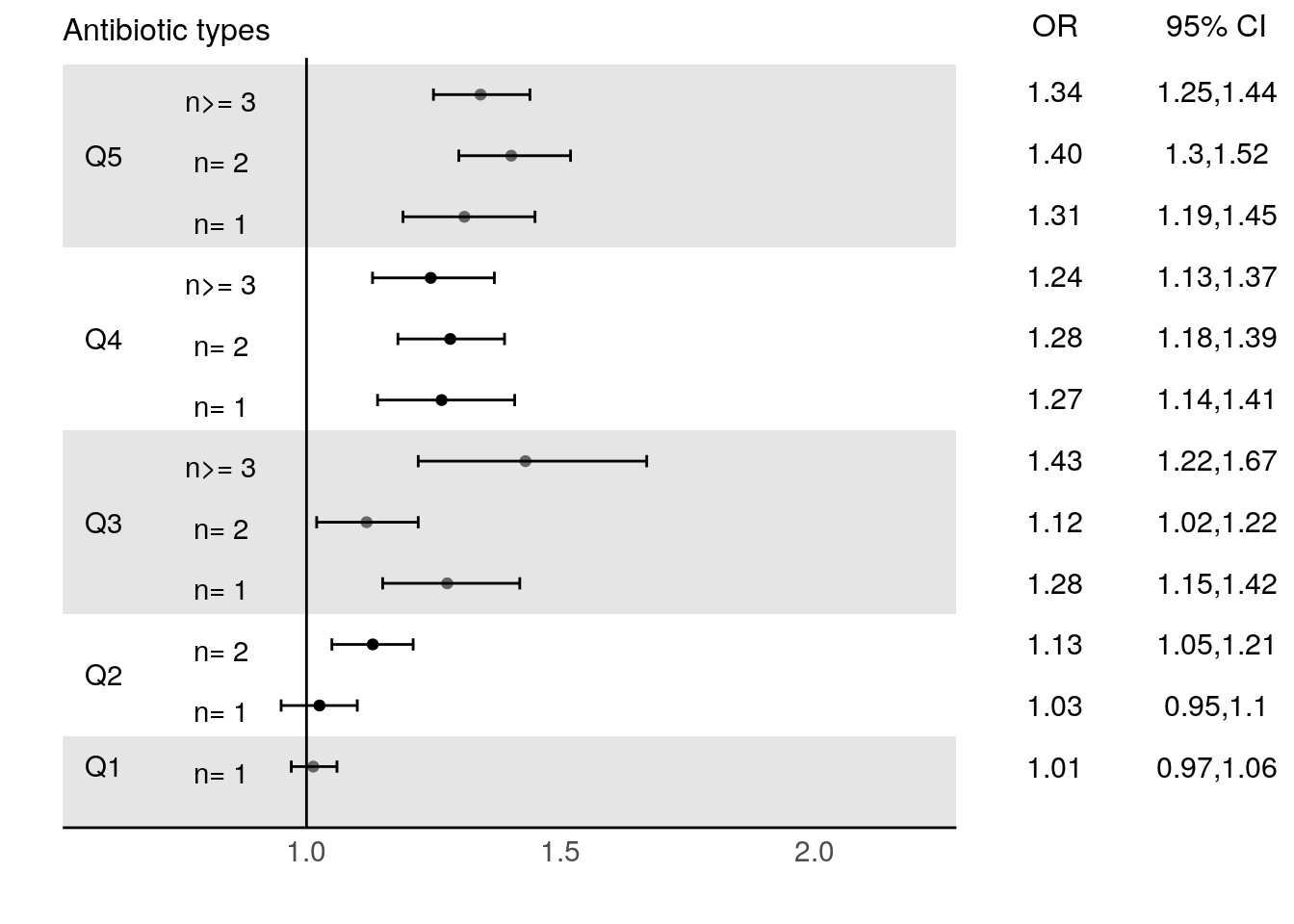 |

*OR were adjusted for ethnicity, BMI category, CCI group, smoking status, IMD, care home residents, COVID-19 and flu vaccine

* Adjusted ORs for COVID-19 outcomes stratified by number of antibiotic types in the 3 years by quintile (Q1-Q5) of total number of prior antibiotic prescription

## Supplementary Figure 8. Age distribution of matched and non-matched cases

| **A. Study 1: admitted to hospitals** |
| --- |
| 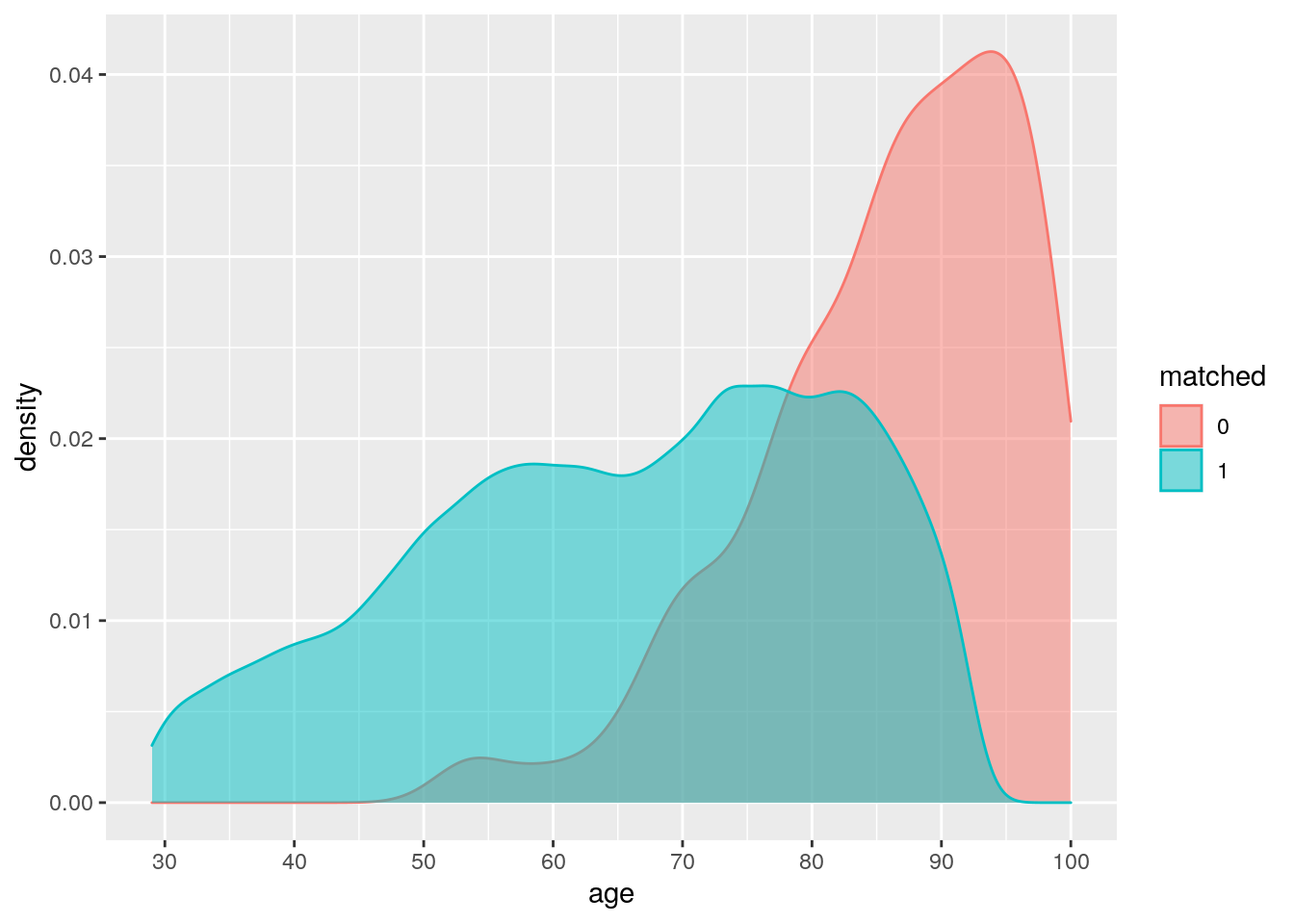 |
| **B. Study 2: death** |
| 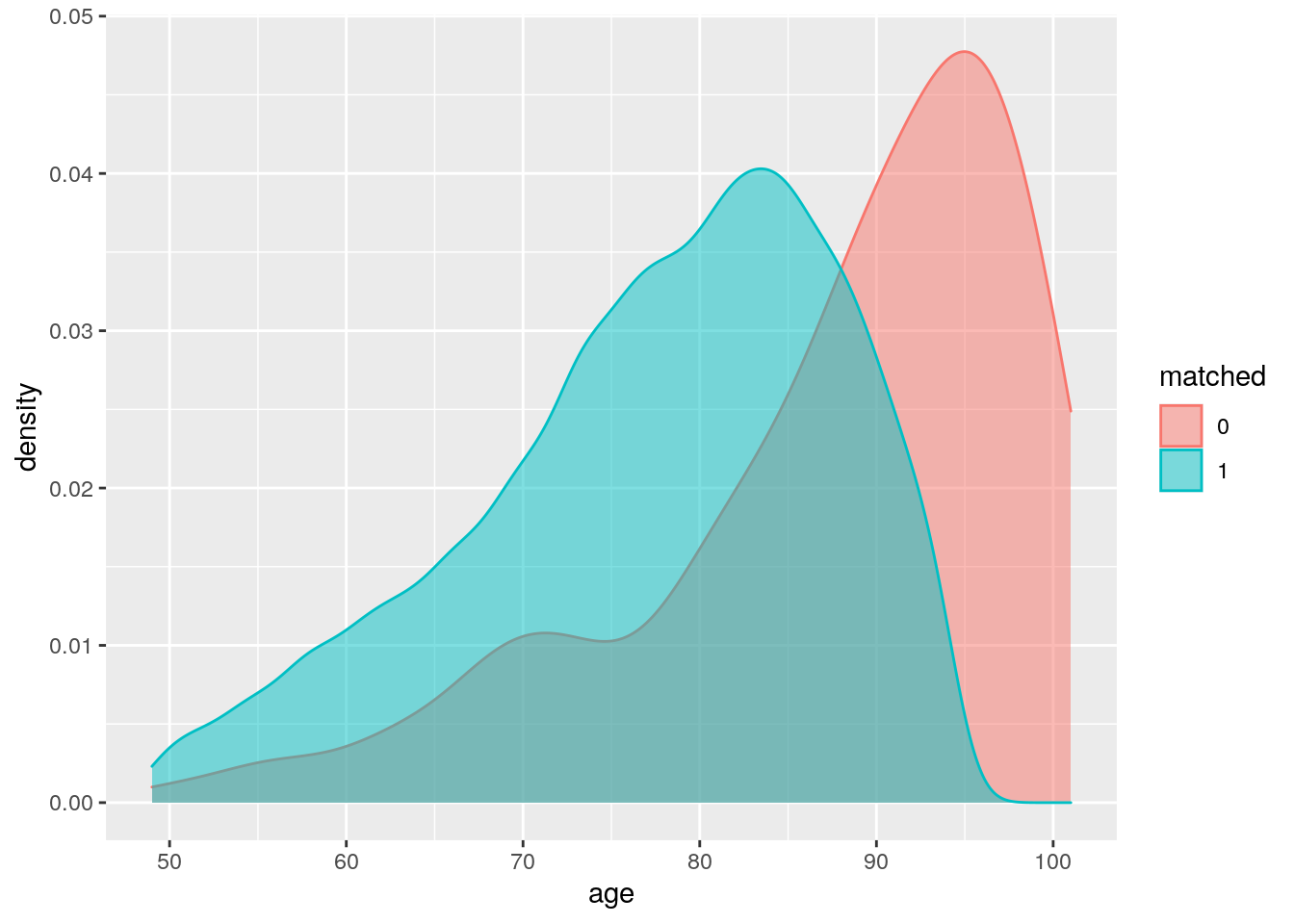 |

## Supplementary Figure 9. Complete case analysis

| **A. Study 1: admitted to hospitals** |
| --- |
| 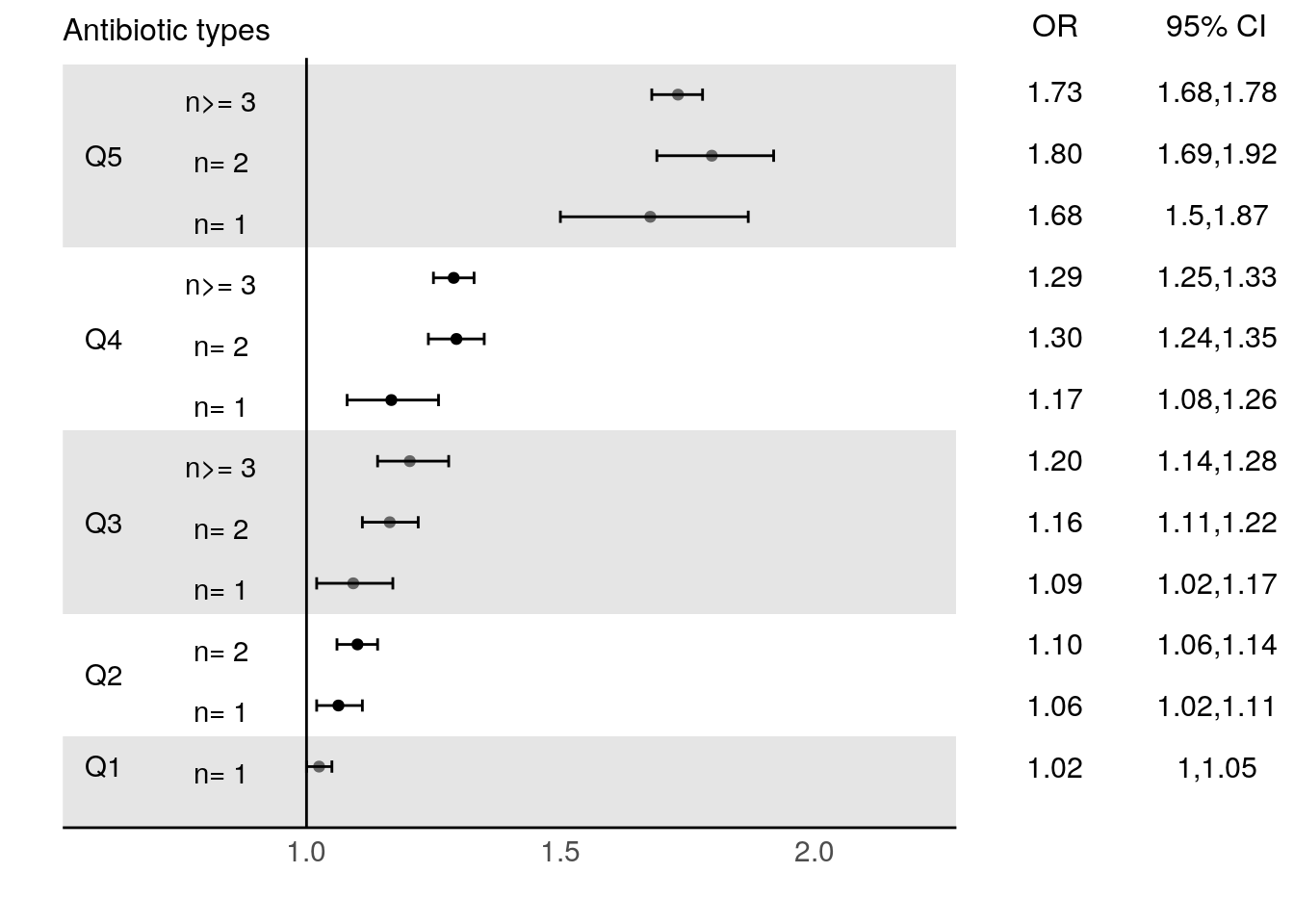 |
| **B. Study 2: death** |
| 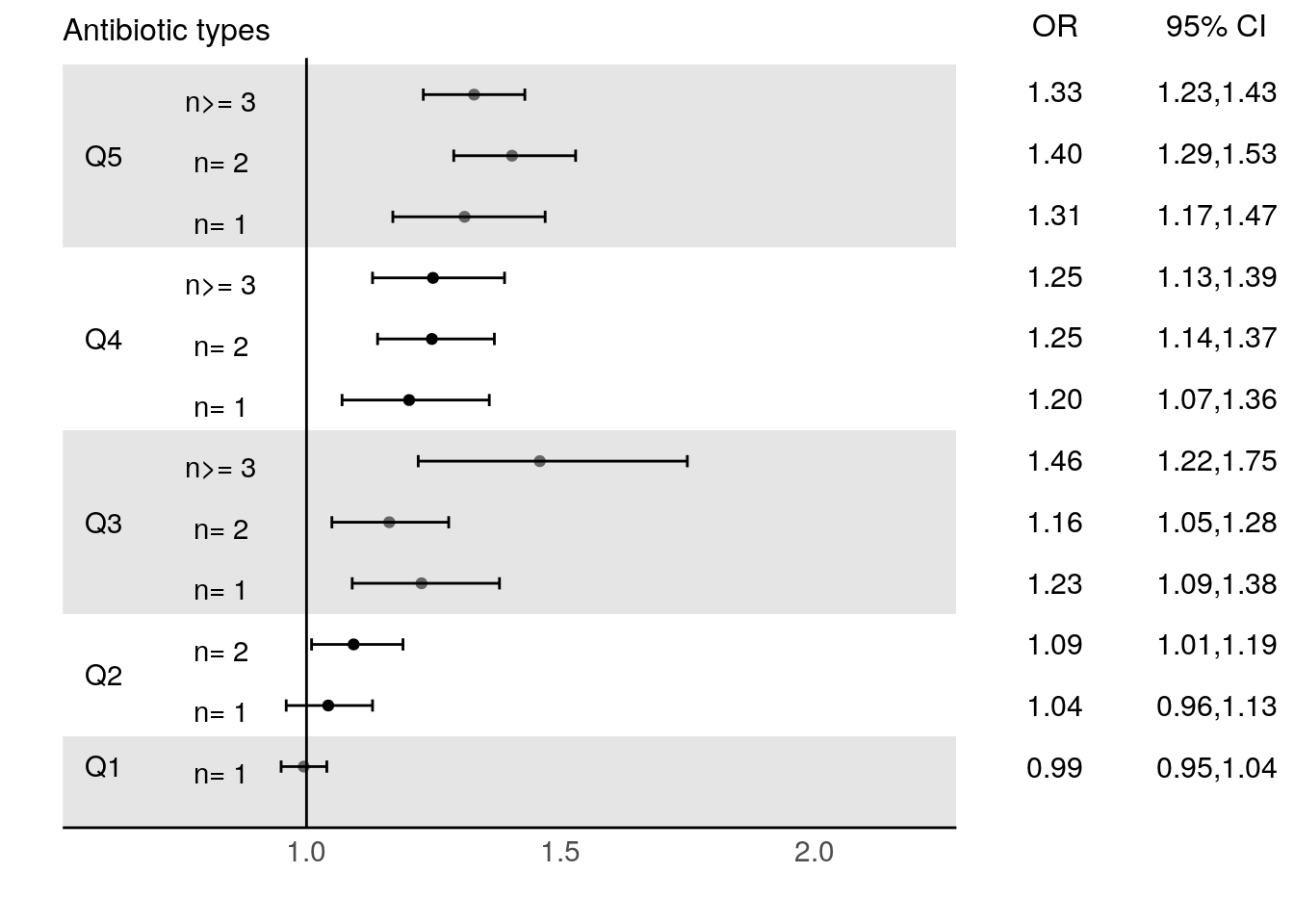 |

*OR were adjusted for ethnicity, BMI category, CCI group, smoking status, IMD, care home residents, COVID-19 and flu vaccine

* Adjusted ORs for COVID-19 outcomes stratified by number of antibiotic types in the 3 years by quintile (Q1-Q5) of total number of prior antibiotic prescription

#

# **Supplementary Protocol**


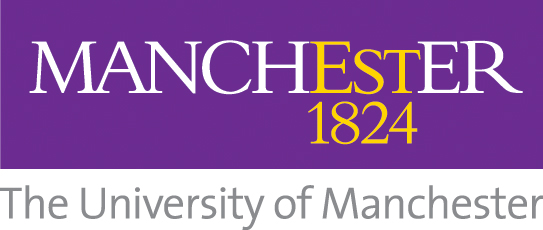


**Exploring the impact of Covid-19 on the primary care treatment pathways for common infections: an analysis with OpenSAFELY platform**

Victoria Palin^1^, Darren M Ashcroft PhD^2^, Tjeerd Pieter van Staa^1^

^1^Centre for Health Informatics & Health Data Research UK North, Division of Informatics, Imaging and Data Science, School of Health Sciences, Faculty of Biology, Medicine and Health, The University of Manchester, Manchester Academic Health Science Centre, Manchester, United Kingdom

^2^Centre for Pharmacoepidemiology and Drug Safety, NIHR Greater Manchester Patient Safety Translational Research Centre, School of Health Sciences, Faculty of Biology, Medicine and Health, The University of Manchester, Oxford Road, Manchester, M13 9PL, UK

Note: this document contains the text as included in the OPENSafely research protocol for the repeat antibiotic analyses. Text related to other objectives is not included.

**INTRODUCTION**

Since the outbreak of Covid-19 in the UK in late January 2020, primary care has changed quite dramatically, with many general practices moving from physical consultations to phone and video consultations.[1,2] This will likely affect monitoring and management of conditions. In addition to this, due to the pressure on NHS resources, there has been a decline in GP referrals (with cancer and mental health services highlighted) in the midst of this pandemic, as well as postponement of some treatments within hospitals (cancer treatments, elective surgeries).[3–6] This in turn, could impact on patient morbidity and mortality.

Antimicrobial resistance (AMR) is an important public health problem which if left unmanaged will lead to significant harm to the population. As an example, Greater Manchester has high levels of antimicrobial resistance compared to other areas in England, yet pre-Covid-19 was in the top 20 areas nationally for low antibiotic prescribing (inappropriate antibiotic prescribing is one of the key drivers of AMR). The vast majority of antibiotics (81%) in the UK are prescribed in primary care.[7] Covid-19 has reduced demand for primary and secondary care services, through a reduction in illness due to reduced social mixing, but possibly because people have been fearful of engaging with services. Both of these factors will have contributed to changes in antibiotic prescribing during the pandemic, however it is as yet unclear if the rate of antibiotic prescribing for the burden of illness experienced went up or down during Covid-19. Covid-19 has also forced changes to clinical practice with a shift to virtual primary care consultations; in the first lockdown approximately 24% of consultations were face to face, compared to more than 70% in the previous year (Royal College of General Practitioners 2021). This could have impacted prescribing in a number of ways. Virtual prescribing may have increased the propensity to prescribe antibiotics as clinicians were not able to physically assess patients. Equally, broad spectrum antibiotic usage could have increased due to the diagnostic uncertainty created by not being able to physically examine a patient. Or, conversely, it is possible that peer pressure on GPs to prescribe antibiotics reduced as patients were not physically in the clinic room. When Covid-19 subsides, it is possible that practitioners (both in primary and secondary care) will retain a higher level of virtual consultations than before the pandemic. It is important to understand how this shift to virtual working has impacted antibiotic prescribing in the context of AMR to inform future prescribing practice.

**Research questions**

The overall objective of this OpenSAFELY project is to inform common infection pathways and chronic disease management activities in primary care (i.e., provide background information without identifying individual practices). Specific aims are to assess:

1. the effects of prior antibiotic exposure (types and extent) on severity of outcomes in Covid-19 infections (including during and after hospital admission).

**METHODS AND ANALYSIS**

**Study population**

A retrospective study will be carried out within OpenSAFELY, a new data analytics platform in England created to address urgent COVID-19 related questions [8]. We will use routinely collected electronic data from primary care practices using TPP SystmOne or EMIS software, covering almost all practices in England, linked to Office of National Statistics (ONS) death registrations, Hospital Episode Statistics (HES) with information on date and cause of hospital admission and Covid-19 laboratory tests. The source population will include about 58 million patients. We will include all patients (any age) alive and under follow-up on 1st February 2020, and with at least one year of continuous GP registration prior to this date, to ensure that baseline data could be adequately captured. We will exclude people with missing age, sex, or index of multiple deprivation, since these are likely to indicate poor data quality [9].

**Statistical methods**

The analyses of the effects of prior antibiotic exposure (types and extent) on severity of outcomes will use logistic regression. These models will include risk factors such as age, sex, calendar year and season, ethnicity, socioeconomic class, comorbidity, record of flu vaccination in the year before, number of non-antibiotic prescriptions in the year before, and hospital referral and inpatient hospitalisation in the year before.[10]

**ADDITIONAL INFORMATION**

The findings from this research will be published in peer-reviewed journals and presented at relevant seminars and conferences. Following publication, any findings of interest to a wider audience will be published in non-peer reviewed patient and professional publications. The results of the infection sub-study will also inform the content of the dashboards (for practices) and Knowledge Support System in the following ongoing research project approved by ethics committee: Knowledge support to General Practitioners and patients: evaluation of the effectiveness of periodic feedback, decision support during consultations and peer comparisons in multi-arm cluster randomised trial (BRIT2); IRAS project ID: 290050; REC reference: 21/NE/0103; Sponsor: University of Manchester). It will be used to inform (i) antibiotic stewardship activities disseminated through our PHE and NICE collaborators, (ii) the dashboards and Decision Support System in the BRIT2 cluster trial, (iii) updated dashboards which provide publicly available information on antibiotic prescribing in primary care (https://www.britanalytics.uk ).

**INFORMATION GOVERNANCE**

OpenSAFELY is a secure platform for analysis of anonymised (de-identified) electronic patient health records. The main purpose of OpenSAFELY is to conduct research in the public interest to deepen our understanding of the Covid-19 -https://docs.OpenSAFELY.org//. To date, 22 studies have been published that used OpenSAFELY ([https://www.OpenSAFELY.org/research/](https://www.opensafely.org/research/)). The common law duty of confidentiality for accessing record level data within the OpenSAFELY platform has been set aside under the COVID-19 COPI regulation 3 notice, which sets aside the duty of confidentiality. < Coronavirus (COVID-19): notification to

organisations to share information - GOV.UK (www.gov.uk)> Therefore, consent for secondary analysis of de-identified patient records is not required for this study. OpenSAFELY has received ethics approval for their research studies. All data will remain within the OpenSAFELY research platform and access is only granted to members of the research team once the required training has been completed and approved by OpenSAFELY. Large scale computational analysis will be performed within the data centre of which, the platform maintains extremely high standards for data privacy whilst ensuring complete computational and analytical transparency. OpenSAFELY has detailed governance procedures and active Oversight Board ([https://www.OpenSAFELY.org/governance/](https://www.opensafely.org/governance/)}. All members of the research team will have up-to-date accredited safe researcher training (SRT).

**SAFETY CONSIDERATIONS AND ADVERSE EVENTS**

The study only concerns data analyses.

**PEER REVIEW**

This project has been submitted to the NHS Ethics Committee due to section 251 involvement in the COPI notice (which forms the legal basis for research use of OpenSAFELY data); this project has previously undergone review by the University ethics committee with favourable opinion.

**ETHICAL AND REGULATORY CONSIDERATIONS**

Research Ethics Committee approval will be obtained before commencing research. The study will be conducted in full conformance with all relevant legal requirements and the principles of the Declaration of Helsinki, Good Clinical Practice (GCP) and the UK Policy Framework for Health and Social Care Research 2017. There will no risks to patients related to this research.

**STATEMENT OF INDEMNITY**

The University has insurance available in respect of research involving human subjects that provides cover for legal liabilities arising from its actions or those of its staff or supervised students. The University also has insurance available that provides compensation for non-negligent harm to research subjects occasioned in circumstances that are under the control of the University.

**FUNDING and RESOURCES**

This work is supported by the independently funded grant by the National Institute for Health Research (HS&DR Project: NIHR130581) and concerns the cluster randomised trial to improve antibiotic prescribing in primary care. The work in this project will inform the dashboards as being used in the previously approved BRIT 2 study.

**PUBLICATION POLICY**

Dissemination of research outputs to participating clinical staff and policy and guideline developers will be an integral part of this project. In addition, we will promote dissemination to national policy makers, managers and clinical leaders, through project summaries and policy briefings. Through our partners in PHE and NICE, we will engage NHS England. We will continue to work closely with Wessex Academic Health Science Network (AHSN) which is the national lead for the AHSNs’ Medicines Optimisation Programme and works collaboratively with the other 14 AHSNs to develop, share and spread good practice.

**PATIENT AND PUBLIC INVOLVEMENT**

We have recruited a patient advisory group of 5+ members via the NIHR Patient research ambassador programme. The advisory group has a lay Chair and will meet quarterly and provides input to shape key study components and analysis and dissemination throughout the multiple work streams. Members of our PPPIE advisory group will also work with us to engage with relevant wider public and community groups including older people and carers in order to raise awareness about responsible antibiotic prescribing and use, and to ensure public perspectives feed into the project and development of appropriate outputs. We will work with community-based groups and conduct community engagement events in participating regions during the project. We will also include at least one PPI representatives on our Study Steering Committee.

**COMPETING INTERESTS**

The authors have no competing interests.

**REFERENCES**

1 Thornton J. Covid-19: how coronavirus will change the face of general practice forever. *BMJ* 2020;**368**:m1279. doi:10.1136/bmj.m1279

2 Appointments in General Practice - March 2020. NHS Digit. 2020.

3 Thomas R. Major drop off in referrals to children’s mental health services. Health Serv. J. 2020.

4 Hiom S. How coronavirus is impacting cancer services in the UK. Cancer Res. UK. 2020.

5 NHS England and NHS Improvement. NEXT STEPS ON NHS RESPONSE TO COVID-19. 2020.

6 Lai AG, Pasea L, Banerjee A, *et al.* Estimating excess mortality in people with cancer and multimorbidity in the COVID-19 emergency. *Preprint* doi:10.13140/RG.2.2.34254.82242

7 NJ Z, M M, CAM M, *et al.* Trends in Antibiotic Prescribing in Out-of-Hours Primary Care in England from January 2016 to June 2020 to Understand Behaviours during the First Wave of COVID-19. *Antibiot (Basel, Switzerland)* 2021;**10**:1–10. doi:10.3390/ANTIBIOTICS10010032

8 OpenSAFELY Collaborative T, Williamson E, Walker AJ, *et al.* OpenSAFELY: factors associated with COVID-19-related hospital death in the linked electronic health records of 17 million adult NHS patients. doi:10.1101/2020.05.06.20092999

9 Bhaskaran K, Bacon S, Evans SJ, *et al.* Factors associated with deaths due to COVID-19 versus other causes: population-based cohort analysis of UK primary care data and linked national death registrations within the OpenSAFELY platform. *Lancet Reg Heal Eur* 2021;**6**:100109. doi:10.1016/J.LANEPE.2021.100109

10 Van Staa TP, Palin V, Li Y, *et al.* The effectiveness of frequent antibiotic use in reducing the risk of infection-related hospital admissions: Results from two large population-based cohorts. *BMC Med* 2020;**18**:40. doi:10.1186/s12916-020-1504-5
